# PIM kinases as therapeutic targets against advanced melanoma

**DOI:** 10.18632/oncotarget.10703

**Published:** 2016-07-19

**Authors:** Batool Shannan, Andrea Watters, Quan Chen, Stefan Mollin, Markus Dörr, Eric Meggers, Xiaowei Xu, Phyllis A. Gimotty, Michela Perego, Ling Li, Joseph Benci, Clemens Krepler, Patricia Brafford, Jie Zhang, Zhi Wei, Gao Zhang, Qin Liu, Xiangfan Yin, Katherine L. Nathanson, Meenhard Herlyn, Adina Vultur

**Affiliations:** ^1^ Program of Cellular and Molecular Oncogenesis, Melanoma Research Center, The Wistar Institute, Philadelphia, PA, USA; ^2^ Department of Dermatology, University Hospital Essen, Essen, Germany; ^3^ Department of Chemistry, University of Marburg, Marburg, Germany; ^4^ Abramson Cancer Center, University of Pennsylvania Perelman School of Medicine, Philadelphia, PA, USA; ^5^ Department of Biostatistics and Epidemiology, University of Pennsylvania School of Medicine, Philadelphia, PA, USA; ^6^ Department of Computer Science, New Jersey Institute of Technology, Newark, NJ, USA

**Keywords:** melanoma, PIM kinases, SGI-1776, organometallics, therapy

## Abstract

Therapeutic strategies for the treatment of metastatic melanoma show encouraging results in the clinic; however, not all patients respond equally and tumor resistance still poses a challenge. To identify novel therapeutic targets for melanoma, we screened a panel of structurally diverse organometallic inhibitors against human-derived normal and melanoma cells. We observed that a compound that targets PIM kinases (a family of Ser/Thr kinases) preferentially inhibited melanoma cell proliferation, invasion, and viability in adherent and three-dimensional (3D) melanoma models. Assessment of tumor tissue from melanoma patients showed that PIM kinases are expressed in pre- and post-treatment tumors, suggesting PIM kinases as promising targets in the clinic. Using knockdown studies, we showed that PIM1 contributes to melanoma cell proliferation and tumor growth *in vivo*; however, the presence of PIM2 and PIM3 could also influence the outcome. The inhibition of all PIM isoforms using SGI-1776 (a clinically-available PIM inhibitor) reduced melanoma proliferation and survival in preclinical models of melanoma. This was potentiated in the presence of the BRAF inhibitor PLX4720 and in the presence of PI3K inhibitors. Our findings suggest that PIM inhibitors provide promising additions to the targeted therapies available to melanoma patients.

## INTRODUCTION

Our knowledge of melanoma pathobiology and genetics has greatly advanced in recent years leading to increasingly effective clinical treatments. These include targeted therapies and immunotherapies, both of which contribute to the regression of advanced disease; however, these are not curative for many patients [1]. Treatment combinations are also available, but such approaches can also lead to resistance or toxicity [2, 3]. Therefore, our arsenal of therapeutic options, while encouraging, still requires improvement and expansion. Many melanoma small molecule inhibitors focus on controlling MAPK signaling due to the high number of mutations found in this pathway and its propensity to become reactivated following treatment [4–7]. However, the contribution of other signaling networks to melanoma progression and therapy resistance is unraveling; such pathways include but are not limited to PI3K/mTOR, signal transducers and activators of transcription (STATs), and nuclear factor kappa-B (NFκB) [5, 8–11]. There is often crosstalk and shared signaling among these pathways; therefore, these can provide noteworthy therapeutic targets. One example features PIM kinases, which were demonstrated to contribute to the progression of several human cancers and for which well-tolerated inhibitors are available clinically [12].

The PIM (provirus integration site for moloney murine leukemia virus) family of serine/threonine kinases is composed of three isoforms, PIM1, PIM2, and PIM3. These are reported to be mainly expressed in hematopoietic, vascular smooth muscle, epithelial, and embryonic stem cells, where they are tightly controlled; however, under pathological conditions, inappropriate overexpression can contribute to malignancy [13]. Unlike other kinases, PIMs are constitutively activated but they are regulated by transcription, translation, and proteosomal degradation, i.e. PIM kinase activity depends on protein levels to control cell survival, growth, and cancer progression [12, 14]. While mechanisms regulating PIM kinase levels are still being unraveled, *Pim1* gene expression is controlled by multiple transcription factors and pathways of relevance to melanoma. For example, STAT3 and STAT5 can bind directly to the *Pim1* promoter following stimulation from growth factors, hormones, and cytokines [15]. Hypoxia can induce PIM1 expression in a hypoxia-inducible factor1α (HIF1α)-independent manner, which can contribute to solid tumor pathobiology and chemoresistance [16, 17]. NFκB was also shown to increase PIM1 expression; for example, inhibiting NFκB activation in B cells impaired CD40-based increases in PIM1 protein levels [18].

MAPK signaling can also be regulated by PIM kinase activity; for example, bone marrow cells with PIM1 depletion or inhibition display impaired ERK phosphorylation [19]. In addition, both the PI3K/AKT and PIM signaling pathways converge to control translation via phosphorylation of eukaryotic translation initiation factor 4E binding protein 1 (4EBP1) as well as to decrease apoptosis by the phosphorylation of BAD [12]. PIM kinases have overlapping activity with AKT in that they share common substrates and they both control apoptosis, cell-cycle progression and metabolism [14]; it has also been suggested that PIM kinases contribute to AKT downstream signaling [20, 21]. Other PIM kinase substrates include but are not limited to p21cip1/waf1, p27 Kip1, CDC25, MYC, MYB, SOCS1/3, MAP3K5 [12], which control cellular proliferation. Thus, PIM kinases provide appealing targets for pharmacological inhibition as they play an integral part of multiple signaling pathways involved in malignancy.

PIM kinases' involvement in cell survival and tumorigenesis was originally demonstrated by their ability to suppress myc-induced apoptosis in mouse models of lymphoma [22]. In fact, overexpression of PIM1 and MYC in the lymphoid compartment of transgenic mice provided a strong oncogenic collaboration resulting in lymphoma *in utero* [22]. The oncogenic capacity of PIM kinases also increases with higher expression levels. On the other hand, knockout of all 3 *pim* genes in mice generates a mild phenotype, indicating favorable toxicity profiles for compounds inhibiting one or multiple PIM isoforms [12]. Adding to this therapeutic advantage, the structure of the ATP-binding pocket of the PIM kinase active site is different from that of other protein kinases, which allows for increased specificity [23]. Thus, the contribution of PIM kinases in tumorigenesis and the capacity to selectively inhibit them with limited toxicity, highlights a potential target for melanoma that has not yet been fully explored.

Here, we present findings from a screen of structurally distinct organometallic kinase inhibitors that identified PIM kinases as promising melanoma targets. We show that PIM kinases are expressed in melanoma patients' samples and cell lines, and that PIM1 inhibition by knockdown studies or the use of a clinically available PIM kinase inhibitor can reduce proliferation, viability, and invasion in preclinical models of melanoma. Moreover, we show that the combination of BRAF and PIM inhibitors impedes tumor growth *in vivo.* Given that AKT and PIM kinases share signaling effectors, we finally explore the advantages of combining PI3K and PIM inhibitors in preclinical models of melanoma.

## RESULTS

### Identification of a novel melanoma-selective kinase inhibitor

**Figure 1:**
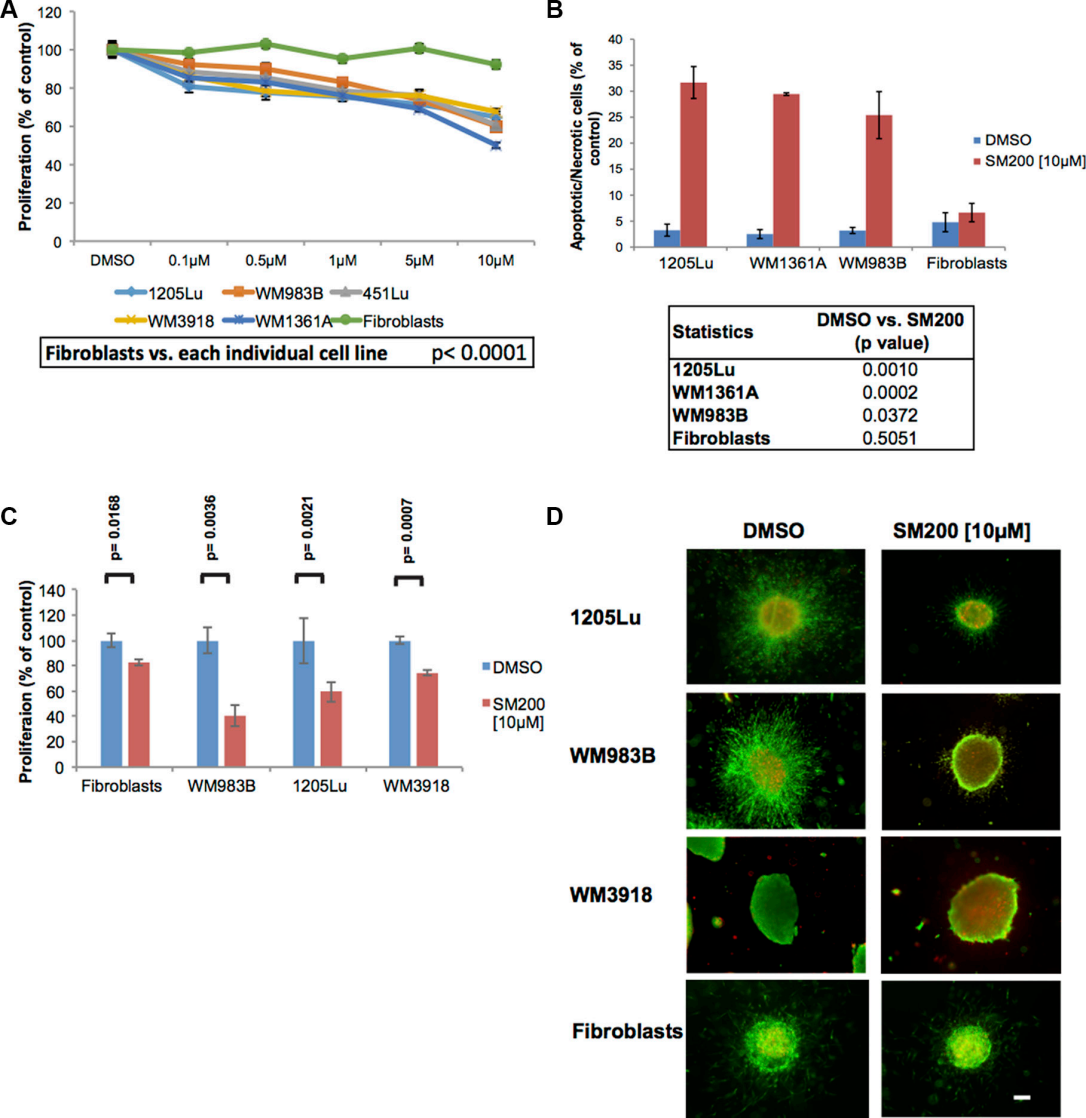
SM200 inhibits proliferation and invasion of 2D and 3D melanoma cells (**A**) Melanoma cells and normal fibroblasts were treated with increasing doses of SM200 for 72 h, then were assessed using the alamarBlue assay. Results were normalized to the DMSO control. Data are represented as mean +/− SEM. A significant response to SM200 was detected for all melanoma cell lines compared to the fibroblasts (*p* < 0.0001). (**B**) Melanoma cell lines and normal fibroblasts were treated with SM200 [10 μM] for 72 h before staining with propidium iodide. All treated melanoma cell lines show a significant increase in cytotoxicity, but not fibroblasts. Data are represented as mean +/− SEM, and *p*-values are provided in the table below. (**C**) The AlamarBlue assay was conducted on collagen-embedded melanoma spheroids treated with SM200 for 72 h to assess spheroid growth. Data are represented as mean +/− SEM, from triplicate experiments and *p*-values are provided. (**D**) Collagen-embedded melanoma spheroids were treated for 72 h with SM200 [10 μM] and stained for live and dead cells. Green fluorescence indicates metabolically active (live) cells, red fluorescence indicates membrane compromised (dead) cells. Experiments were conducted in triplicate and representative images are shown. Scale bar represents 150 microns.

Organometallic compounds, compared to other small molecule inhibitors, offer properties such as increased structural diversity, adjustable ligand exchange kinetics, fine-tuned redox activities, and distinct spectroscopic signatures, which make them highly versatile for the regulation, sensing, and imaging of biological processes [24]. We designed 34 novel inert metal-containing compounds that serve as highly potent and selective inhibitors of protein kinases and lipid kinases [25] and evaluated them for their anti-melanoma activity (compound structures available in the supplementary information). These compounds were used to treat normal skin-derived fibroblasts and a panel of genetically diverse human-derived melanoma cell lines (Supplementary Table S1) over 72 h using the MTS assay. The goal was to identify compounds with melanoma inhibitory properties but minimal effects on normal cells such as fibroblasts. Most compounds tested were ineffective in reducing melanoma cell line proliferation, some were cytotoxic to all cells, or displayed an IC_50_ above 10 μM (Supplementary Table S2). However, we observed three compounds that slowed proliferation in melanoma cell lines at doses of 10 μM or below but not in normal fibroblasts. This effect was most pronounced for SM200 across multiple melanoma cell lines and this was validated using the alamarBlue assay (Figure 1A). We next examined if SM200 was anti-proliferative or cytotoxic. Results from a propidium iodide assay show that SM200 causes significant cell death in melanoma cell lines but not in fibroblasts (Figure 1B). We did not detect high levels of caspase-3 staining by FACS analysis; however, 72 h post-treatment may be too late to detect early apoptotic events (Supplementary Figure S1).

Since compounds have been shown to fail in more complex culture models and even induce adverse effects in some contexts, we evaluated SM200 in three-dimensional (3D) melanoma spheroids embedded in a collagen matrix [8]; these more readily mimic the *in vivo* milieu [26]. Quantitation of collagen-embedded spheroid response to SM200 using the alamarBlue assay showed a significant decrease in metabolic activity in the 3D context (*p* < 0.005 for all melanoma lines tested; Figure 1C). We next observed that SM200 displayed anti-invasive and cytotoxic properties in 3D using a LIVE/DEAD viability/cytotoxicity assay, while the effects on normal fibroblasts were less pronounced (Figure 1D). Thus, SM200 was further evaluated for its anti-melanoma activity.

### The anti-melanoma inhibitor SM200 inhibits PIM kinases

**Figure 2:**
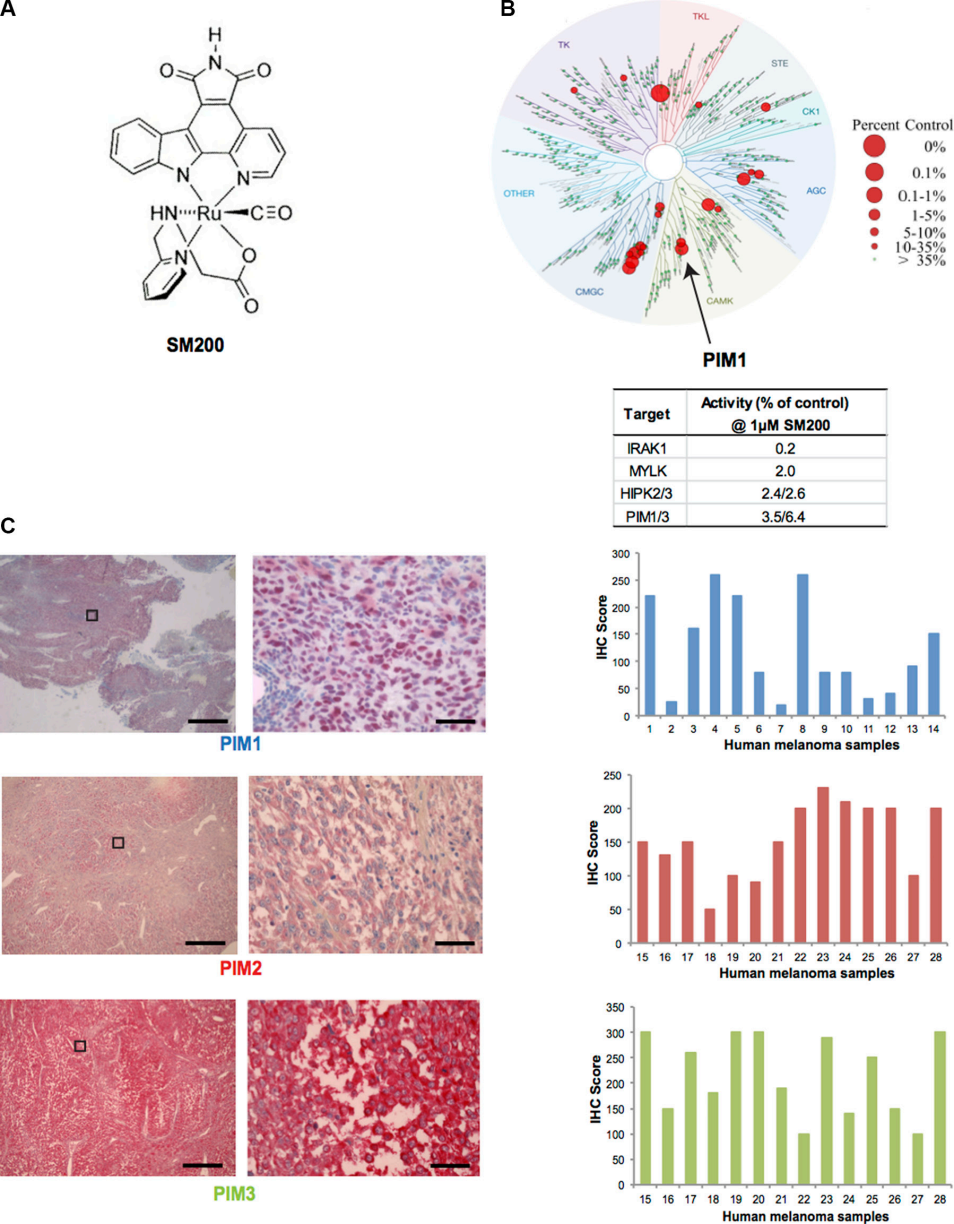
PIM kinases as targets for melanoma (**A**) Chemical structure of SM200. The compound was used as a racemic mixture but only one enantiomer is shown. (**B**) Human kinome phylogenetic tree (TREEspot, DiscoveRx) displaying SM200 selectivity as assessed by an active-site-directed affinity screening approach against 451 human protein kinases (KINOMEScan). The human kinase dendogram shows the protein kinase families and the evolutionary relationships between the individual kinases. Compound hits are shown as red dots (%ctrl = percent of control: 0% = highest affinity drug binding, 100% = no affinity drug binding); PIM1 is indicated with an arrow. SM200 was profiled for protein kinase binding at a concentration of 1 μM and the top four inhibited kinases are shown in the right hand table. (**C**) Images of PIM1, PIM2, and PIM3 staining (IHC) of human metastatic melanoma tissue is shown. The images on the right correspond to a higher magnification of the images on left (boxed area). Scale bars represent 50 μM (left images) and 1 μM (right images). PIM1, PIM2, PIM3 expression was interpreted for fourteen different samples using the H-score method. Images for sample #4 (PIM1) and #17 (PIM2 and PIM3) are provided. The intensity and extent of staining on the entire tissue sections were assessed according to a four-tiered (0 to 3) scale.

SM200 is a ruthenium-containing organometallic complex with a structure that was inspired by the natural product staurosporine (Figure 2A) [27]. Interestingly, staurosporine was previously shown to display anti-melanoma activity [28]. SM200 is inert (stable) and does not undergo any ligand exchange so that all interactions between the organometallic compound and protein kinases are mediated through the ligand sphere. The pyridocarbazole moiety is designed to hydrogen bond with the hinge region of protein kinases, whereas the remaining coordination sphere forms contacts with other parts of the ATP-binding site. In particular, co-crystal structures of related organometallic complexes with protein kinases revealed that the CO ligand forms important interactions with the flexible glycine-rich loop [29].

To gain insight into the protein kinase inhibition properties of SM200, we tested its protein kinase binding affinity profile at 1 μM against the majority of the human protein kinases encoded in the human genome (human kinome) [30]. This was accomplished using an active-site-directed competition binding assay with 451 different protein kinases (KINOMEscan, DiscoveRx) which provides primary data that correlate with binding constants (Kd) [31, 32]. The main SM200 kinase hits identified were IRAK1, MYLK, HIPK1-3, PIM1 and PRKG2 (at values below 4% of controls); PIM3 was also inhibited (6.4% of controls) (Figure 2B, Supplementary Table S3). Given the important role of PIM kinases in malignancies as well as the availability of PIM kinase inhibitors for use in the clinic, we decided to investigate the role of PIM kinases in melanoma pathobiology and therapy [14, 28].

To confirm PIM kinases as valid targets for melanoma patients and to assess expression variability, we stained human melanoma tissue for PIM1, PIM2, PIM3. (PIM kinase staining of normal skin is best shown by the Human Protein Atlas (http://www.proteinatlas.org). While PIM staining for all three isoforms was present in all tumor samples, intensity varied as assessed using the H-Score method, suggesting that some patients may be more sensitive to PIM inhibition than others (Figure 2C). No tumor sample displayed low levels of all three PIM isoforms.

### Contribution of PIM kinases to melanoma proliferation

**Figure 3:**
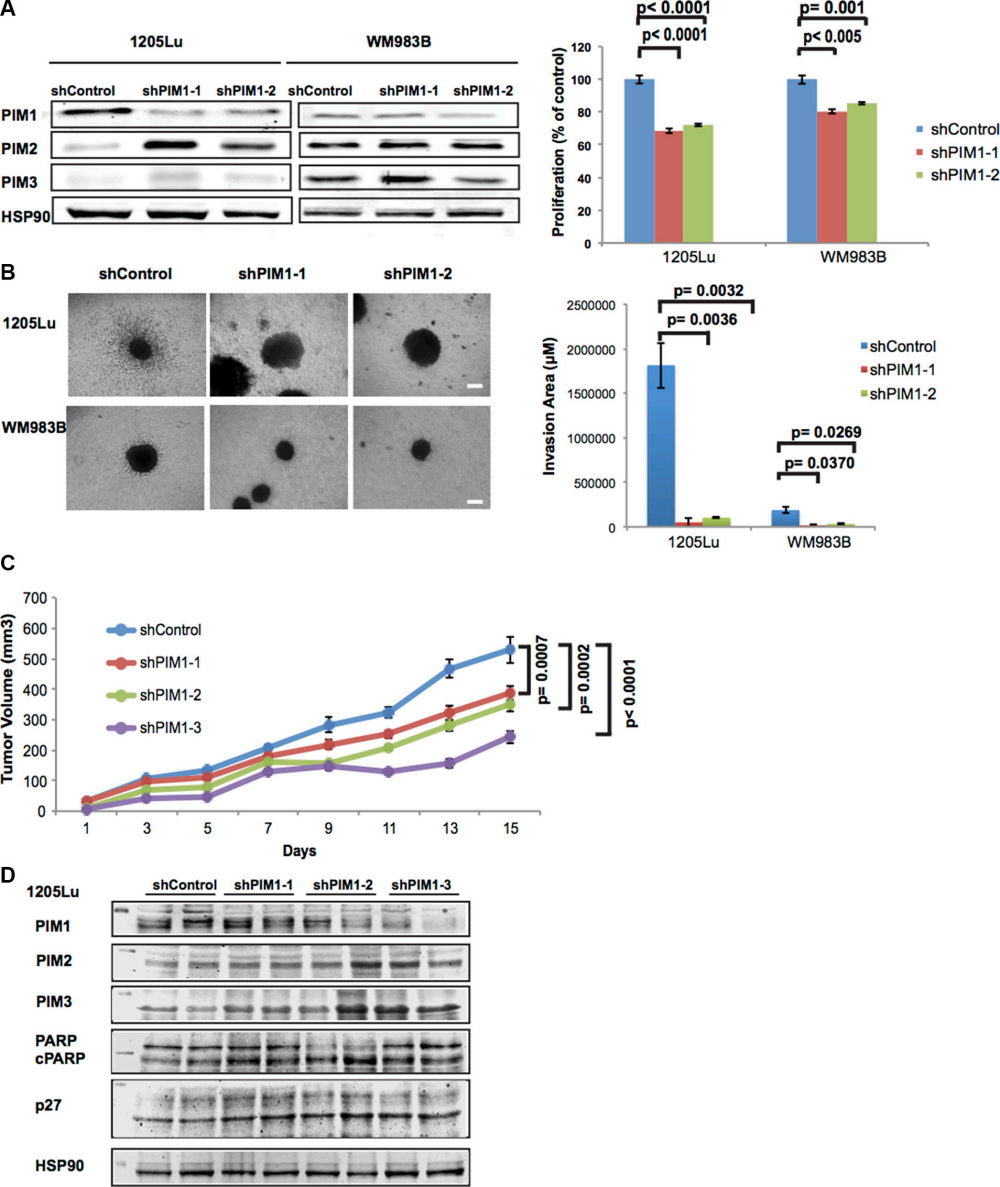
Effects of PIM1 knockdown on melanoma cells (**A**) Left panel: PIM1 was knocked down in 1205Lu and WM983B melanoma cells using two different shRNAs; expression of PIM isoforms was then assessed by western blot. Right panel: MTS assay shows decreased proliferation of melanoma cells with PIM1 knockdown. Data are represented as mean +/− SEM, from triplicate experiments. (**B**) Left panel: Phase contrast images of 3D spheroids generated with PIM1 knockdown melanoma cells. Scale bar represents 150 microns. Right panel: Quantitation of invasion from spheroids generated with PIM1 knockdown cells. Data are represented as mean +/− SEM, from three separate spheroids. (**C**) NSG mice were xenotransplanted using 1205Lu melanoma cells with PIM1 knocked down using 3 different shRNAs. Tumor volumes were measured at the indicated time points until day 15. Statistical analyses of tumor growth rates used ANOVA with groups defined by each shRNA. Three comparisons are shown for each shRNA group versus controls (*p* < 0.001 for all comparisons). Error bars represent SEM, *n* = 8 mice/group. (**D**) Western blot analyses of tumor lysates from PIM1 knockdown 1205Lu xenotransplanted mice (2 different tumors/group). Levels of all three PIM kinases are shown (below the 50 kDa marker) as well as p27 (below 35 kDa marker). Cleaved PARP and the loading control Hsp90 are also shown (below 100 kDa marker).

To determine if knocking down (KD) PIM1 activity is sufficient to reduce melanoma proliferation, we used two distinct PIM1-inhibiting shRNAs. Both shRNAs reduced PIM1 levels and this was most evident in 1205Lu cells, which also displayed higher levels of PIM1 in the parental cell line (Figure 3A). The knock down was repeated and confirmed earlier observations (Supplementary Figure S2). An MTS assay showed that all cell lines with PIM1 KD displayed decreased metabolic activity indicative of reduced proliferation compared to the empty vector controls. Cells with reduced PIM1 levels showed compensatory upregulation of other PIM kinase isoforms but this was not observed in all cases. To further explore the effects of knocking down PIM1 in melanoma cells, we cultured the PIM1 KD cells as 3D spheroids and observed decreased invasion compared to the empty vector controls (Figure 3B).

Given the anti-proliferative and anti-invasive effects of PIM1 KD on melanoma cells grown *in vitro*, we conducted *in vivo* studies to confirm the role of PIM1 in melanoma tumor growth. We injected PIM1KD-1205Lu cells featuring three separate shRNAs in NOD-SCID-IL2-γ-null (NSG) mice, and monitored tumor growth for a period of 15 days (Figure 3C). We observed that 1205Lu shPIM1 significantly reduced tumor growth (*p* < 0.001 for all shRNAs used). Upon further investigation using western blot analysis of tumor samples, PIM1 expression was reduced in most shPIM1 tumors, while levels of p27 and cleaved PARP were elevated. We also observed elevated levels of PIM2 and PIM3, possibly as a compensatory mechanism to offset the lack of PIM1 (Figure 3D). Figure 3A and Supplementary Figure S2 also show the upregulation of PIM2 following PIM1 knockdown; however, additional cell lines need to be investigated to more fully understand the compensatory effects between PIM isoforms. Our findings suggest the benefit of inhibiting the PIM1 kinase in melanoma and also the need to investigate the contribution of the other PIM isoforms in melanoma biology.

### PIM kinase inhibition in melanoma using a clinically-relevant PIM kinase inhibitor

**Figure 4:**
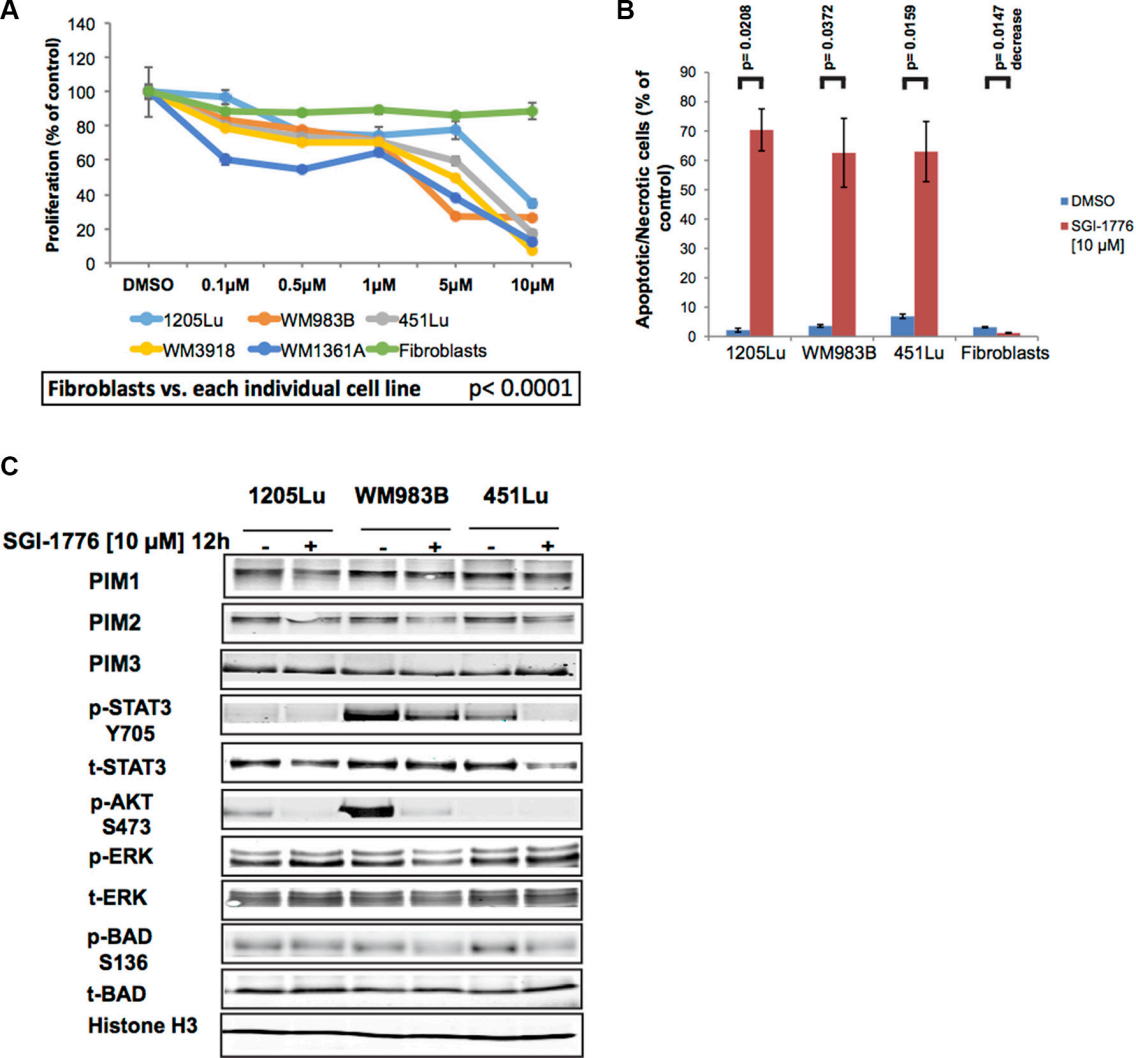
SGI-1776 inhibits proliferation and invasion of melanoma cells (**A**) Melanoma cells and normal fibroblasts were treated with increasing doses of SGI-1776 for 72 h and were assessed using the alamarBlue assay. Results were normalized to the DMSO control. Data are represented as mean +/− SEM. A significant response to SGI-1776 was detected for all melanoma cell lines compared to the fibroblasts (*p* < 0.0001). (**B**) Three melanoma cell lines and normal fibroblasts were treated with SGI-1776 [10 μM] for 72 h before staining with propidium iodide. All treated melanoma cell lines show a significant increase in cytotoxicity, but not fibroblasts. Data are represented as mean +/− SEM. (**C**) Western blot analyses of adherent (2D) melanoma cells treated with SGI-1776 [10 μM] for 12 h. Levels of all PIM isoforms were investigated as well as multiple effectors involved in PIM signaling. Histone H3 was used as a loading control.

Since SM200 is an organometallic compound with no current application in the clinic, we examined the PIM kinase inhibitor SGI-1776, which has previously been investigated in patients. SGI-1776 is a drug candidate imidazo[1,2-b]pyridazine that inhibits all three PIM kinases with IC_50_ values of 7 nM, 363 nM, and 69 nM for PIM1, −2 and −3, respectively [33]. Treatment of AML xenografts with this pan-PIM kinase inhibitor was previously shown to cause concentration-dependent tumor regressions [34]; therefore, we investigated the effects of this drug on different preclinical models of melanoma. Using adherent melanoma cultures and the alamarBlue assay, we observed that SGI-1776 had inhibitory effects similar to that of SM200 in that melanoma cell lines were most inhibited but not normal fibroblasts (*p* < 0.0001 for all cell lines analyzed) (Figure 4A). SGI-1776 [10 μM] also significantly increased cell death in the melanoma cell lines compared to normal fibroblasts using propidium iodide staining (*p* < 0.05 for all cell lines analyzed; Figure 4B).

We next explored the signaling changes that accompany SGI-1776 treatment in three melanoma cell lines, 1205Lu, WM983B, and 451Lu. Western blot analyses indicate that SGI-1776 does not dramatically reduce total levels of PIM1, −2, or −3, a possible outcome if feedback loops are affected or if drug-induced protein degradation occurs. However, consistent with the expected effects of the drug on downstream PIM kinase signaling effectors, downregulation of pBADS136, pSTAT3Y705, and pAKTS473 levels were observed (Figure 4C). The collective data thus suggest that PIM kinase inhibition using SGI-1776 has anti-proliferative and cytotoxic effects in preclinical models of melanoma, and that SGI-1776 downregulates PIM-related signaling effectors that are also involved in melanoma pathobiology.

### SGI-1776 displays anti-tumor activity in combination with BRAF inhibition

**Figure 5:**
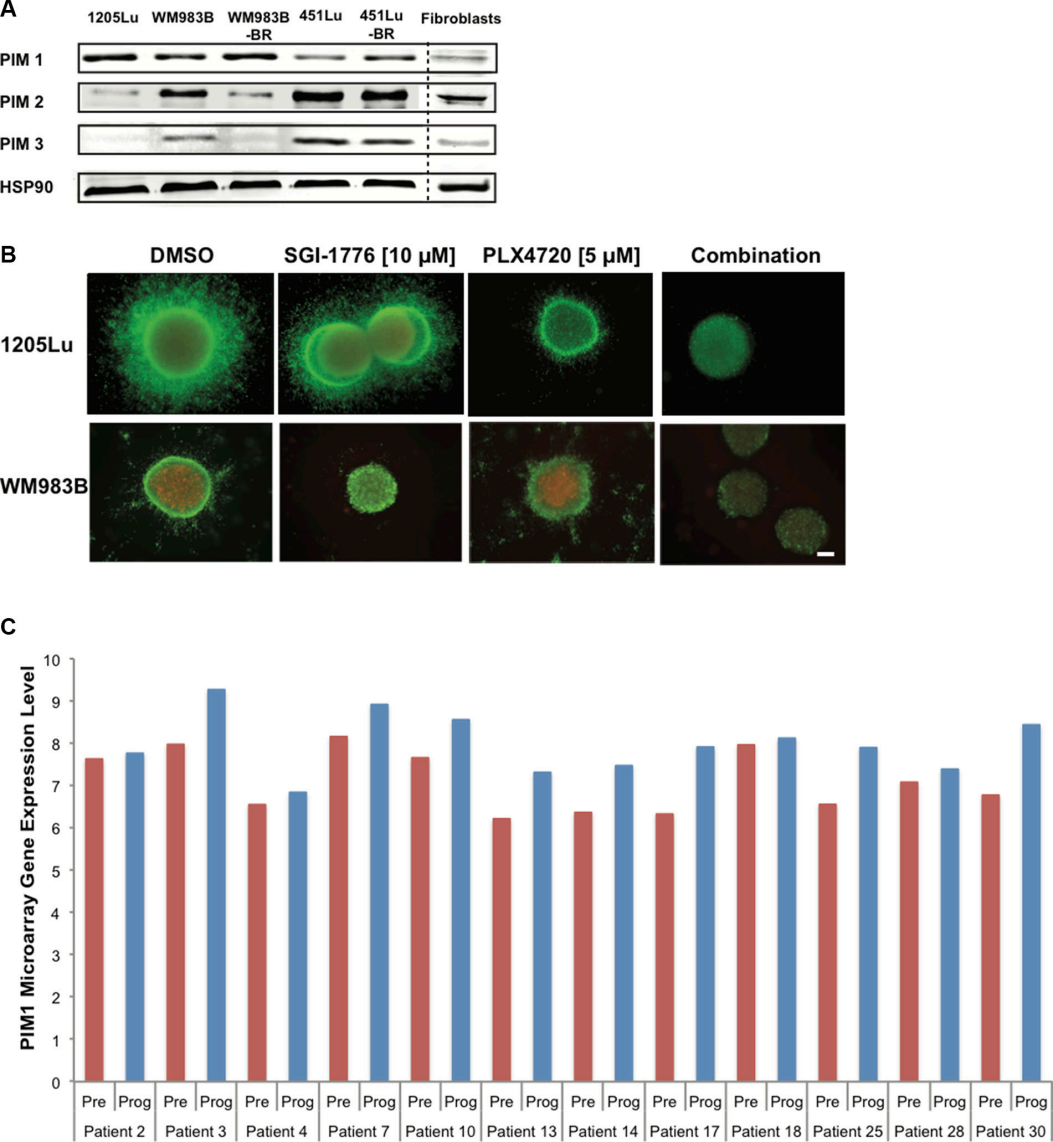
SGI-1776 displays anti-tumor activity in combination with a BRAF inhibitor in 3D melanoma models (**A**) Western blot analysis showing endogenous levels of PIM-1, −2, and −3 in human melanoma cell lines, normal fibroblasts, and cell lines rendered resistant to BRAF inhibitors (BR). Hsp90 served as loading control. (**B**) Collagen-embedded melanoma spheroids were treated for 72 h with SGI-1776 [10 μM] or PLX4720 [5 μM] as single agents or in combination. Spheroids were then stained for live (green) and dead (red) cells. Experiments were conducted in triplicate and representative images are shown. Scale bar represents 150 microns. (**C**) PIM1 gene expression in melanoma patient samples pre-treatment and upon treatment progression on dabrafenib or vemurafenib. Total RNA was isolated from fresh frozen melanoma tumors in 21 patients (GSE50509). The graph shows data for 12 patients with greater PIM1 gene expression in progressing tumor samples (Prog) than in pretreated samples (Pre). The red and blue bars represent the normalized microarray expression of PIM1 (on a log scale) in Pre and Prog samples, respectively.

Currently, patients with advanced melanoma are given immunotherapies and/or targeted therapies (often against the MAPK pathway) to curb disease. Therefore, any new potential inhibitor with clinical relevance is likely to be paired with standard treatments or to be given to patients with resistant tumors. We thus investigated endogenous levels of PIM1, −2, and −3 in three mutant BRAF melanoma cell lines as well as cell lines rendered resistant to BRAF inhibitors (BR) (generated and characterized in [5]); fibroblasts were used as controls. We observed the expression of PIM1 and PIM2 in all melanoma samples, while PIM2 and PIM3 levels were most elevated in samples expressing lower PIM1 levels (Figure 5A). Fibroblasts had low levels of all three PIM isoforms. Our results suggest that PIM inhibitors could be useful for pre- and post-BRAF inhibitor treatment melanomas, it also supports observations from Figure 2C. We next examined if the effects of SGI-1776 in melanoma preclinical models could be further enhanced in the presence of a BRAF inhibitor. When mutant BRAF melanoma cells were grown as 3D spheroids embedded in collagen and were stained with the LIVE/DEAD assay, SGI-1776 [10 μM] as a single agent reduced cell invasion and growth, and this was potentiated in the presence of the BRAF inhibitor PLX4720 (Figure 5B).

To verify if PIM kinase inhibition could be relevant to patients displaying drug resistance to current targeted therapies, we analyzed RNAseq data from melanoma patient tumor samples. These samples were isolated pre-treatment and upon progressive disease, following single agent BRAF inhibition or BRAF and MEK inhibitor combination treatment. Our analyses indicate that compared to the pre-treatment sample counterparts, PIM1 levels are found elevated in approximately half of the patients displaying progressive disease, more specifically in 12/21 paired samples in one dataset (GSE50509, [35]) (Figure 5C) and 4/9 paired samples in an additional dataset (GSE61992 [36], Supplementary Figure S3). These results are in accordance with the elevated PIM1 levels also detected in our BRAF inhibitor resistant cell lines. Our observations thus suggest that PIM1 could be a beneficial target in tumors resistant to current MAPK inhibitors.

### The PIM inhibitor SGI-1776 displays anti-melanoma effects *in vivo*

**Figure 6:**
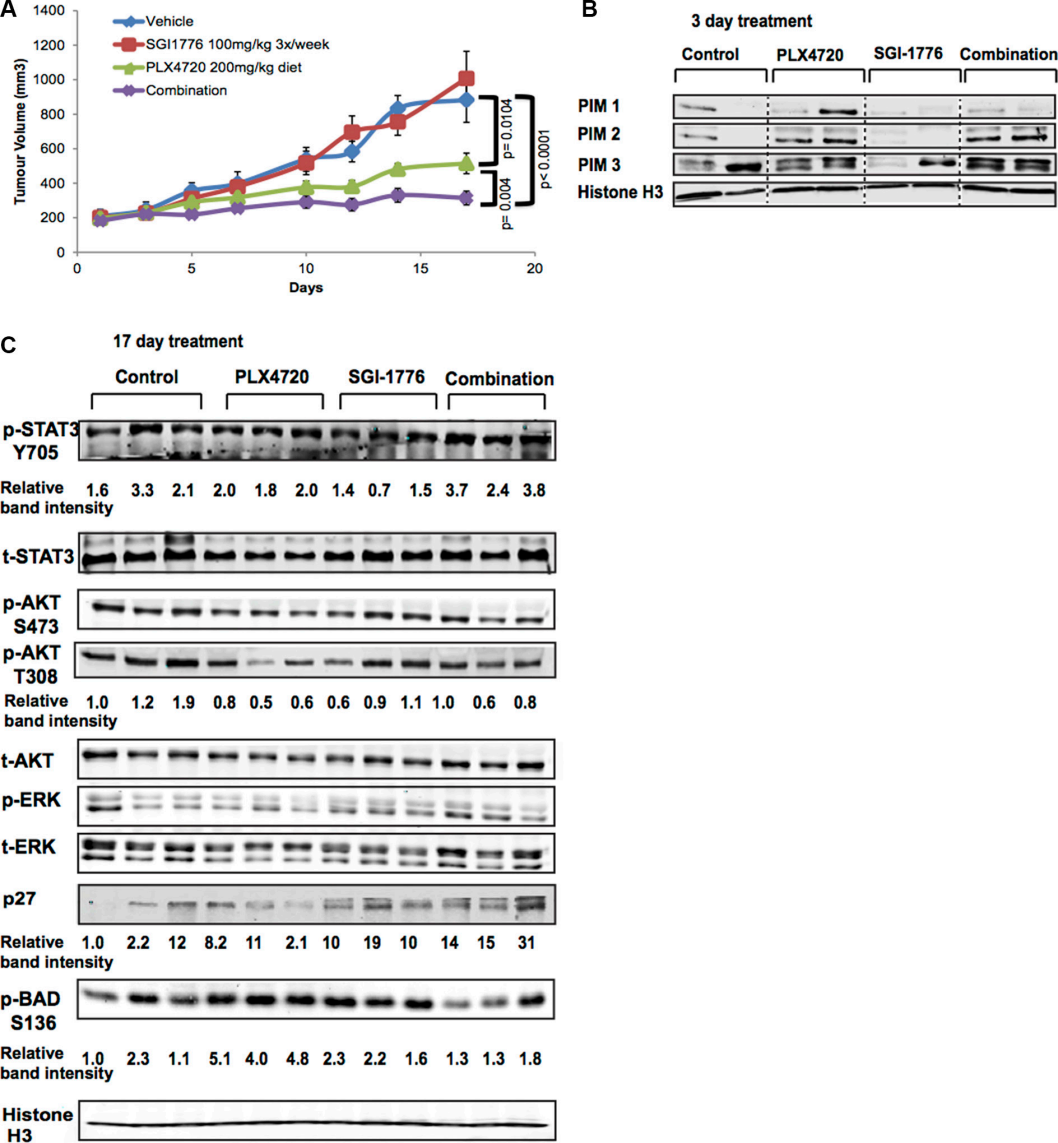
SGI-1776 displays anti-tumor activity in combination with a BRAF inhibitor *in vivo* (**A**) NSG mice were xenotransplanted with 1205Lu melanoma cells and tumors were allowed to grow above 200 mm^3^. Mice were then treated with the single agents SGI-1776 (100 mg/kg 3×/week), or PLX4720 (200 mg/kg diet), or with the combination of both drugs. Tumor volumes were measured at the indicated time points. Statistical analyses of tumor volumes used one-way ANOVA with groups defined by treatment (*p* < 0.001 for all comparisons shown). Error bars represent SEM, *n* = 10 mice. (**B**) PIM kinase expression (all three isoforms) in mouse tumor lysates (*n* = 2/group); Histone H3 serves as a loading control. Lysates were collected 3 days after treatment initiation. (**C**) Western blot analyses of mouse tumor lysates (*n* = 3/group) isolated on day 17 of the experiment shown in (A). Effectors of the PI3K, MAPK, and STAT3 signaling pathways, as well as pBAD and the tumor suppressor p27, were evaluated. Histone H3 served as loading control.

Since we demonstrated that SGI-1776 displays anti-melanoma effects in 3D melanoma models, we investigated the therapeutic value of this agent *in vivo* using a mutant BRAF model of melanoma. BRAF inhibitors are currently used in the clinic against mutant BRAF melanomas; thus, we tested whether a combination strategy of SGI-1776 and the BRAF inhibitor PLX4720 could potentiate an anti-tumor response in a xenograft model featuring *BRAF* mutant 1205Lu cells, which are marginally responsive to PLX4720 [5]. We injected 1205Lu cells in NOD-SCID-IL2-γ-null (NSG) mice, then initiated treatment once 200 mm^3^ tumors were established. We used doses that would not cause complete tumor growth arrest from the single agents in order to assess their combinatorial potential. As shown in Figure 6A, treatment of tumor-bearing mice with the single agents did not fully inhibit 1205Lu tumor progression; however, with a combination of PLX4720 (daily diet) and SGI-1776 (3 times/week oral dosing), significant tumor growth arrest was observed without causing obvious toxic effects (Supplementary Figure S4A). SGI-1776 was also effective in reducing tumor growth as a single agent using higher drug doses (Supplementary Figure S4B); however, this can lead to body weight loss following constant treatment beyond two weeks [34].

We next determined the expression levels of PIM1, −2 and −3 in two mouse tumors from the *in vivo* combination experiment shown in Figure 6A. Tumor samples were collected following 3 days of treatment and PIM expression levels were not consistently changed across treatment groups; however, the presence of PLX4720 correlated with higher PIM2 levels (Figure 6B). To understand signaling changes leading to our observed drug response *in vivo,* western blot analyses were then conducted on tumor lysates isolated 17 days following treatment (Figure 6C). We observed elevated p27Kip1 levels in SGI-1776 treated samples, with the highest levels detected in the slowest growing SGI-1776+PLX4720 treated tumors. These combination-treated tumors also had lower levels of pBADS136. However, we did not observe dramatic changes in phosphorylated AKT or STAT3 (as seen in Figure 4C). Our findings suggest that SGI-1776 displays anti-melanoma effects in combination with BRAF inhibition *in vivo*; however, the inactivation of pathways such as PI3K and/or STAT3 may be necessary to obtain tumor regressions.

### Dual PIM and PI3K inhibition inhibit melanoma cell survival

**Figure 7:**
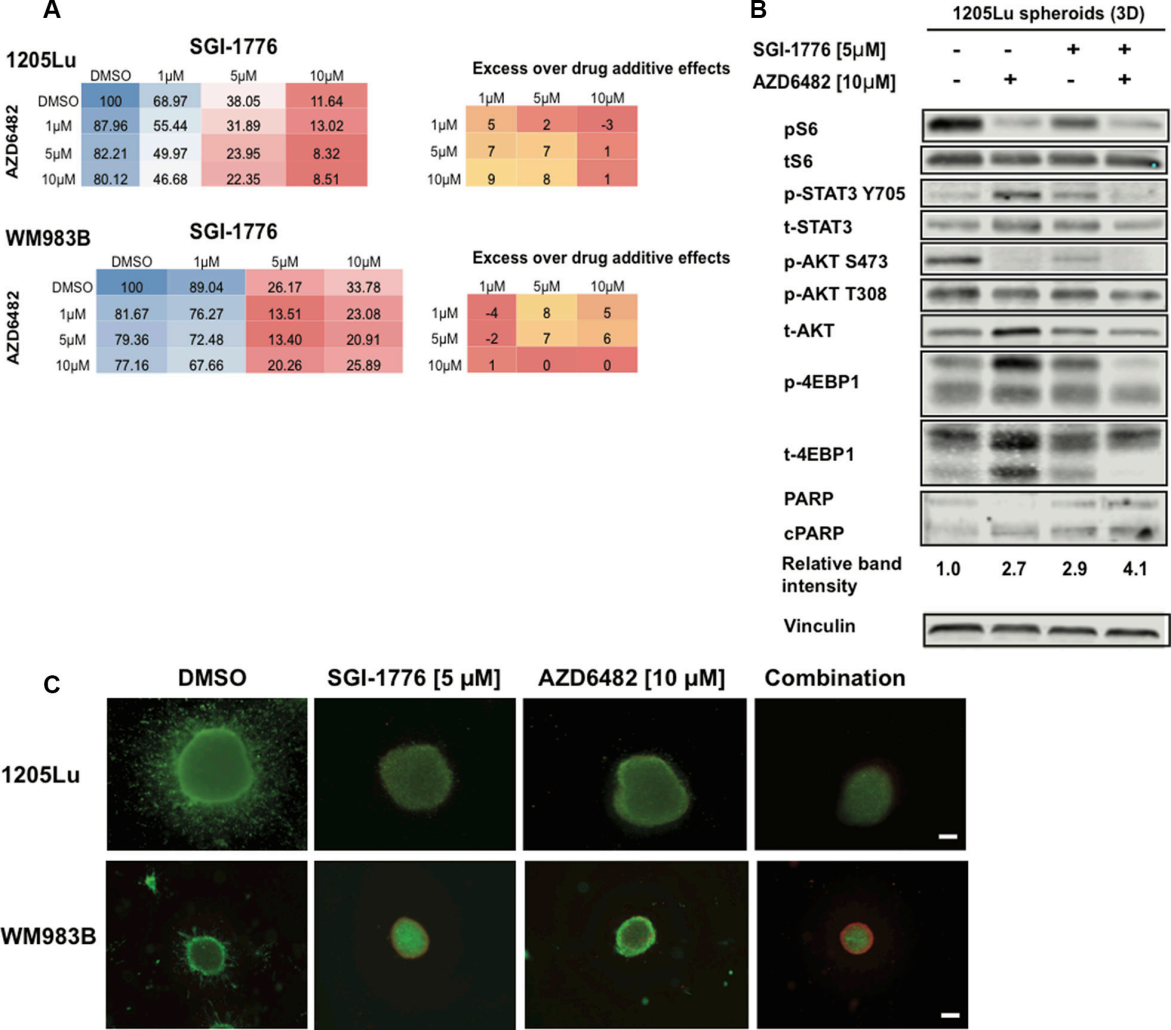
SGI-1776 displays anti-tumor activity in combination with a PI3K inhibitor in 3D melanoma models (**A**) Melanoma cells were treated with increasing doses of SGI-1776 or AZD6482 for 72 h and were assessed using the alamarBlue assay. The mean % of proliferation compared to the DMSO control is shown (from three experiments). Blue indicates no inhibition, red indicates high inhibition. Synergy was calculated for combination experiments using the Bliss formula. The Bliss number gives the difference between predicted and observed inhibition values (excess over Bliss); a positive value indicates synergy, a negative value indicates antagonism and values near zero indicate an overlap of predicted and observed combination effects. (**B**) 1205Lu spheroid lysates were interrogated for effectors of AKT and STAT3 signaling, as well as cleaved PARP by western blots. Vinculin was used as a loading control. (**C**) Collagen-embedded melanoma spheroids were treated for 72 h with SGI-1776 [5 μM] or AZD6482 [10 μM] as single agents or in combination before staining for live (green) and dead (red) cells. Experiments were conducted in triplicate and representative images are shown. Scale bar represents 150 microns.

Since PIM kinases and AKT share common downstream effectors, we hypothesized that inhibiting both PIM and PI3K activity would prevent pathway compensation, enhance melanoma cell death compared to each single agent, and maintain low activity of AKT and STAT3. We thus used adherent melanoma cultures, the alamarBlue assay, SGI-1776, and the PI3Kβ inhibitor AZD6482 in a grid-like design of constant ratio drug combinations in order to assess drug combination effects and facilitate the analysis of synergy. Doses of AZD6482 were selected based on western blot analyses confirming AKT inhibition (Supplementary Figure S5). Synergy was observed for the SGI-1776 and AZD6482 combination using the Bliss formula [37]; however, this did not occur at the same doses in the two melanoma cell lines investigated (Figure 7A). We note here that observations on synergy are difficult at doses where single agent activity is high as seen for SGI-1776 at 10 μM. While additional cell lines, inhibitors, and concentrations need to be studied, our results suggest that synergy can be achieved with the PI3K/PIM inhibition strategy.

Using our 3D spheroid model of melanoma and western blot analyses, we observed that the combination of the PIM inhibitor SGI-1776 with the PI3Kβ inhibitor AZD6482 caused the lowest levels of pS6, pSTAT3, pAKT, p4EBP1, and increased levels of cleaved PARP (Figure 7B). This widespread inhibition of key effectors involved in melanoma cell survival is not seen for the single agents, suggesting the benefit of inhibiting both PIM and PI3K signals. Using the LIVE/DEAD assay, we confirmed that the single agents could inhibit spheroid invasion and cause cell death, and this was enhanced when both SGI-1776 and AZD6482 are combined (Figure 7C). These data support the benefit of co-inhibiting PI3K signaling and PIM kinases in melanoma. Additional drug combinations of SGI-1776 with the AKT inhibitor MK2206 or the MEK inhibitor UO126 were also studied and suggest synergistic effects between compounds (Supplementary Figure S6); however, confirmation of synergy should be investigated with additional cell lines and drug concentrations. In sum, our results indicate that PIM kinase inhibitors have the potential to enhance the effects of multiple small molecule inhibitors currently available to melanoma patients.

## DISCUSSION

Our approach of evaluating structurally-distinct organometallic compounds to identify melanoma-specific inhibitors led to the identification of a PIM kinase inhibitor (SM200). SM200-related anti-melanoma observations were further explored using PIM1 knockdown studies and a clinically available pan-PIM kinase inhibitor (SGI-1776). Since our results show that PIM1 knockdown can cause a reduction in melanoma cell proliferation and invasion (*in vitro*), or tumor growth *in vivo*, we suggest a contribution of PIM kinases to melanoma pathobiology and the possibility to inhibit such activity. Given that PIM1 knockdown was insufficient to completely halt melanoma cell growth and the expression of different PIM kinase isoforms was still detected, compensation mechanisms between PIM isoforms needs to be further explored especially since not all isoforms share the same effectors [38]. In this respect, inhibitors that target all PIM isoforms are expected to provide more robust therapeutic outcomes.

While PIM kinases are involved in the development and progression of multiple malignancies, little is known about their contribution to melanoma progression [39]. Data from the Cancer Genome Atlas (TCGA) suggest that PIM1 gene alterations (mutations, amplifications, deletions) are found in up to 8% of melanomas, while PIM2 and PIM3 alterations are found in up to 2% and 3% of cases respectively (www.cbioportal.org). Since PIM kinases are constitutively active and regulated at the transcriptional level, pathway deregulation could be underestimated using genetic analyses alone. Indeed, we observed that all our human melanoma samples and cell lines displayed PIM staining (with some isoforms being more predominant than others depending on the sample). Our panel of cell lines also included BRAF inhibitor resistant cells, suggesting a wider potential application for PIM kinase inhibitors for resistant tumors [5]. Interestingly, our analyses of RNAseq data from patients' samples using datasets featuring pre-treatment and treatment progression samples also indicated that tumors progressing on BRAF and MEK targeted therapies show elevated levels of PIM1 compared to pre-treatment samples. Thus, PIM kinase inhibitors could provide a therapeutic option for melanoma patients displaying resistance to targeted therapies.

Clinical trials for PIM kinases as biomarkers or targets for drugs are ongoing and will determine their potential in patients with hematologic malignancies or solid tumors (clinicaltrials.gov; identifiers: NCT02066883, NCT01588548); melanoma patients in the future could benefit from such studies. Because the PIM kinase active site ATP-binding pocket is different from other protein kinases, PIM inhibitors could be more specific than most kinase inhibitors allowing for a better cytotoxic profile, and possibly more favorable therapeutic combinations [12]. The use of a clinically-tested PIM kinase inhibitor SGI-1776 in a melanoma mouse xenograft model in combination with a BRAF inhibitor showed that while we do not dramatically reduce total levels of PIM kinases through this method, there is therapeutic benefit in decreasing their downstream signaling effectors. For example, previous studies demonstrated that SGI-1776 causes significant tumor regressions in animal models of acute myeloid leukemia and suppresses solid tumor growth in models of bladder cancer [34, 40]. We observed that SGI-1776 also prevents melanoma tumor growth *in vivo* as a single agent and when combined with the BRAF inhibitor PLX4720. This suggests that PIM kinase inhibitors could be repurposed for melanoma. While the clinical development of SGI-1776 was discontinued (SuperGen, Inc., Dublin, CA), PIM kinases remain important targets for multiple malignancies and new PIM inhibitors are being developed.

PI3K/AKT signaling is often activated in melanoma [41]. Since PIM and AKT share partially overlapping pathways, residual AKT signaling could interfere with anti- tumor effects. Indeed, previous studies have shown that either expression of a dominant-negative PIM1 or genetic deletion of the kinase increased AKT phosphorylation and expression levels in cardiomyocytes [20]. Our combination of SGI-1776 and the PI3K inhibitor AZD6482 in 3D melanoma models showed that inhibition of AKT signaling is better achieved by the combination treatment; phosphorylated levels of STAT3 were also reduced, highlighting the benefits of inhibiting multiple pathways involved in melanoma resistance [8]. As improved targeted strategies will emerge clinically for PIM kinases and other targeted- and immuno-therapies, new combinations are expected to improve clinical outcomes.

In conclusion, organometallic complexes serve as useful scaffolds to identify novel therapeutic targets. Our approach highlighted PIM kinases as contributors to melanoma pathobiology *in vitro* and *in vivo*. We also provide an expression landscape for PIM kinases across different human melanoma samples. Finally, we present combinatorial strategies featuring a clinically-available PIM kinase inhibitor and MAPK or PI3K-targeting agents. This study adds to our understanding of PIM kinases in melanoma, it highlights a need to consider these along with other pathways involved in melanoma progression, and it also suggests the potential to repurpose PIM inhibitors for melanoma patients.

## MATERIALS AND METHODS

### Cell culture and reagents

Human melanoma cell lines (Supplementary Table S1) were previously described [5, 42, 43]. Cells were cultured in DMEM with 5% fetal bovine serum and grown at 37°C in 5% CO_2_. Normal human fibroblasts were isolated from the dermis of neonatal foreskin [44, 45]. The consistency of cellular genotypes and cell line identities were confirmed by DNA fingerprinting, using Coriell's microsatellite kit. Lentiviral PIM1 shRNA with the pLKO.1 backbone were obtained from OpenBiosystems (Lafayette, CO); shPIM1-1 (TRCN0000010115), shPIM1-2 (TRCN0000010116), shPIM1-3 (TRCN0000010117). Lentiviruses were produced by transfection of 293T cells with the packaging plasmids along with the lentiviral shRNA vector using Lipofectamine 2000 (Invitrogen) according to the manufacturer's protocol. Melanoma cells were exposed to virus in the presence of 8 μg/mL polybrene for 18 h. Knockdown efficiency was determined by western blot analysis for the respective proteins using the anti-PIM1 antibody ab75776 (Abcam, Cambridge, MA).

### Synthetic compounds

All organometallic compounds were synthesized as previously described [46–48]. The synthesis of SM200 is provided as Supplementary Information. PLX4720 was supplied by Plexxikon/Roche (Berkeley, CA); SGI-1776 was purchased from Active Biotech (Lund, Sweden), AZD6482 and MK2206 were purchased from Selleckchem (Houston, TX, USA), and UO126 was purchased from Promega (Madison, WI). All compounds were stored at −20°C in DMSO as 10 mM stocks.

### Proliferation and cell cycle assays

Proliferation was assessed by seeding 5000 cells/well in 96-well plates and allowing cells to adhere overnight. After a 72 h compound treatment, cells were assessed using the CellTiter-96 Aqueous One Solution Cell Proliferation Assay (Promega, Madison, WI), or AlamarBlue (Invitrogen, Grand Island, NY), and absorbance was measured as per the suppliers' instructions. Percent proliferation was normalized to the absorbance of DMSO-treated cells. Cell cycle and apoptosis analyses were conducted on cells grown on 10 cm plates (5 × 10^4^ cells/ml) and drug-treated for 72 h. Cells were trypsinized, fixed with 70% ice-cold ethanol, then stained with propidium iodide (Sigma, St Louis, MO). Cell cycle analyses were conducted using FACSAriaII (Becton Dickinson, Franklin Lakes, NJ) and the CellFIT Cell Cycle Analysis Program (Becton Dickinson).

### Immunoblot analyses

For immunoblots, proteins were extracted as described in [49], and 50 μg of cell extract were resolved on a 10% polyacrylamide-SDS gel before being transferred onto a polyvinylidene membrane (BioRad, Hercules, CA). All primary antibodies were purchased from Cell Signaling Technologies (Beverly, MA), except for the PIM1 antibody, which was purchased from Abcam (Cambridge, MA). All membranes were probed with primary antibodies overnight at 4°C, then incubated with Alexa Fluor-labeled secondary antibodies (IRDye 680LT goat-anti mouse, IRDye 800CW goat-anti rabbit (LI-COR, Lincoln, NE)) for 1h and scanned with the Odyssey system (LI-COR, Lincoln, NE). The secondary antibodies, IRDye700 and IRDye800, and the Odyssey Infrared Imaging System used to image the membranes were obtained from Li-Cor (Lincoln, NE).

### Collagen-embedded melanoma spheroids

Melanoma spheroids were generated according to previous studies [50]. Briefly, 5000 cells/well in 96-well plates were allowed to coalesce on a non-adherent agar layer for 72 h before removal and incorporation in a collagen type I mixture. Spheroids were stained with the Live/Dead cell assay (Invitrogen) then imaged using a Nikon Inverted TE2000 microscope (Melville, NY). Images were analyzed using the ImagePro software (Media Cybernetics, Rockville, MD) and levels of cell death were measured as the average dead signal intensity across spheroids.

### Immunohistochemistry

Twenty-eight cases of metastatic melanoma were randomly selected from the surgical pathology files at the University of Pennsylvania Medical Center. The protocol was approved by the University of Pennsylvania Institutional Review Board. Immunohistochemical assays were performed on formalin-fixed, paraffin-embedded sections. Briefly, 5 μM-thick sections were cut and deparaffinized in xylene and rehydrated in graded alcohols. After antigen retrieval and blocking endogenous peroxidase activity, slides were incubated with 1:100 anti-PIM1 antibody (ab75776), anti-PIM2 antibody (ab118157) or anti-PIM3 antibody (ab71321) for 1h at room temperature (Abcam, Cambridge, MA). Staining was done on a DakoCytomation Autostainer using the EnVision+ horseradish peroxidase (HRP) DAB system (DakoCytomation) according to manufacturer's recommendations. Normal mouse serum (1:1000 dilution) was substituted for the primary antibody as a negative control. Immunohistochemical stains were interpreted semi-quantitatively using the H-score method by assessing the intensity and extent of staining on the entire tissue sections present on the slides according to a four-tiered (0 to 3) scale.

### Kinase screen

The kinase inhibitory profile of SM200 was generated by KINOMEscan (DiscoveRx, San Diego, CA), using an active site directed competition assay to quantitatively determine the interactions between small molecules and 451 protein kinases. Briefly, compounds that bind a kinase active site prevent kinase binding to an immobilized ligand, which reduces the amount of kinase captured on a solid support. Screening “hits” were identified by measuring the amount of kinase captured in control versus compound samples using a quantitative, ultra-sensitive qPCR method that detect associated DNA labels.

### *In vivo* studies

All animal experiments were performed in accordance with The Wistar IACUC protocol 111954 in NOD/LtSscidIL2Rγnull mice (NSG). Mice were each inoculated s.c. with 1 × 10^5^ 1205Lu human melanoma cells in a 1:1 suspension of matrigel (BD Matrigel™ Basement Membrane Matrix, Growth Factor Reduced, Becton Dickinson) and complete media. Drug treatment started at an average tumor volume of 200 mm^3^. Mice were randomized into four groups and treated with: (i) vehicle control, (ii) PLX4720, 200 mg/kg diet, (iii) SGI-1776 every other day (200 mg/kg 3 times a week) or (iv) a combination of PLX4720 and SGI-1776. Hydroxypropyl-β-cyclodextrin (1%) in distilled water (Cyclodextrin Technologies Development, Inc, La Jolla, CA) served as vehicle control. Tumor growth was measured every 2–3 days using a caliper and volumes calculated according to the formula *V = (W* × *D* × *H)/2* [mm^3^]. Tumor samples were snap frozen in liquid nitrogen for subsequent protein analyses or fixed in formalin for histological assessment and IHC staining.

### Gene expression analyses

Two datasets from NCBI Gene Expression Omnibus (GEO) database (http://www.ncbi.nlm.nih.gov/geo/) with ID GSE50509 and GSE61992 were used to analyze the gene expressions of PIM1 in pretreated tumor samples and upon progression. Data were normalized, background-corrected, and summarized using the R package “lumi” [51].

### Statistical analyses

The analysis of variance (ANOVA) or a *t*-test was used to evaluate mean differences between groups. Tukey's procedure was used to compare means when the ANOVA was significant. Levene's test was used to test for equality of variances prior to the ANOVA. When variances were unequal, Welch's ANOVA or t-statistic was used. Error bars are defined in the figure legends.

## SUPPLEMENTARY MATERIALS




