# Supplementary material for: PIM kinases as therapeutic targets against advanced melanoma
Source: Oncotarget. 2016 Jul 19;7(34):54897–912. doi: 10.18632/oncotarget.10703 (PMC5342389; doi:10.18632/oncotarget.10703)
Supplement: Supplementary file 1 [file oncotarget-07-54897-s001.pdf]

PIM kinases as therapeutic targets against advanced melanoma

Supplementary Materials

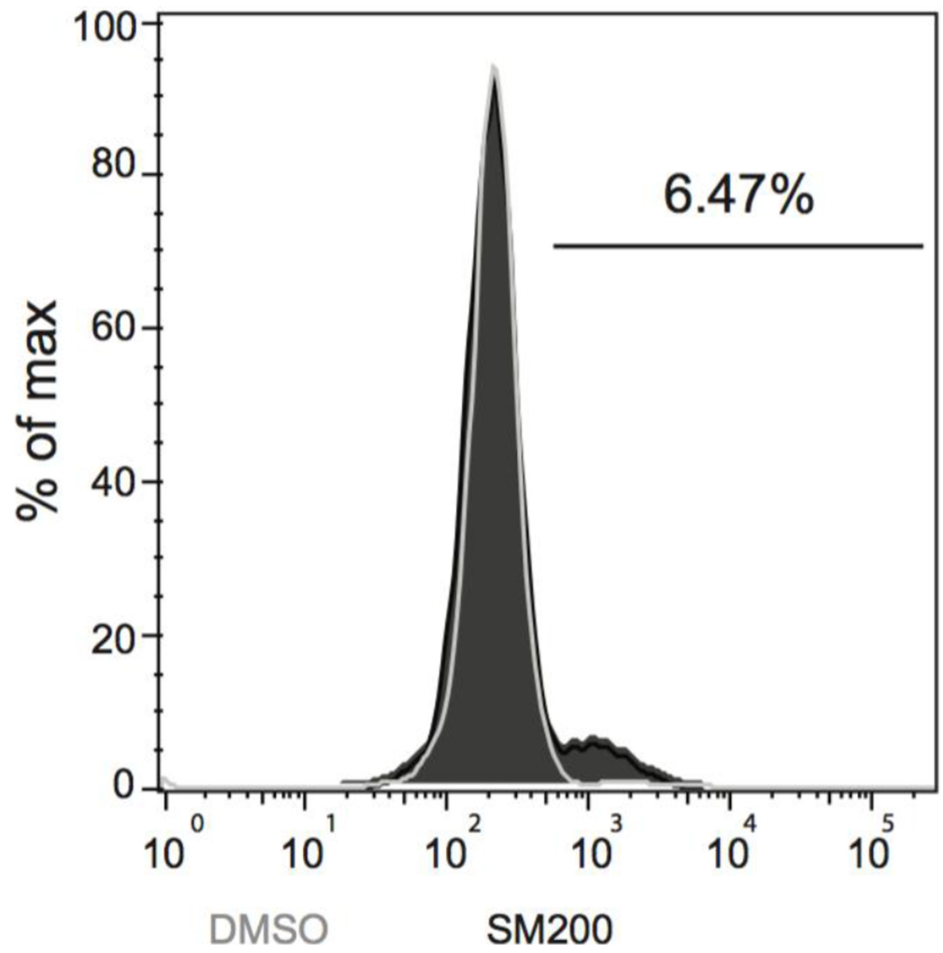

Supplementary Figure S1: Caspase-3 staining following SM200 treatment. Histograms represent caspase-3 activated staining in 1205Lu cells after DMSO (grey line) or SM200 treatment (black filled histogram). The percentage indicates the frequency of caspase3-positive cells found after 72 h SM200 (10 µM) treatment. On X and Y axes, fluorescence intensity and % of max are respectively reported.

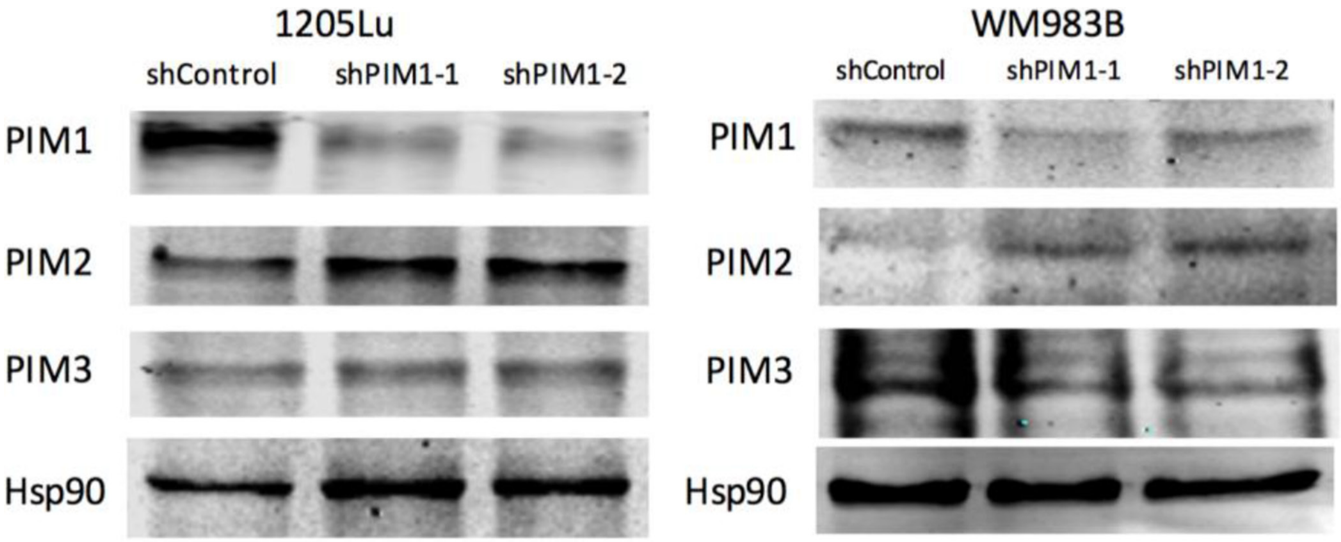

Supplementary Figure S2: Effects of PIM1 knockdown on melanoma cells. PIM1 was knocked down in 1205Lu and WM983B melanoma cells using two different shRNAs; expression of PIM isoforms was then assessed by western blot. These cell lines were independently established and are distinct from those shown in Figure 3A. Hsp90 served as loading control.

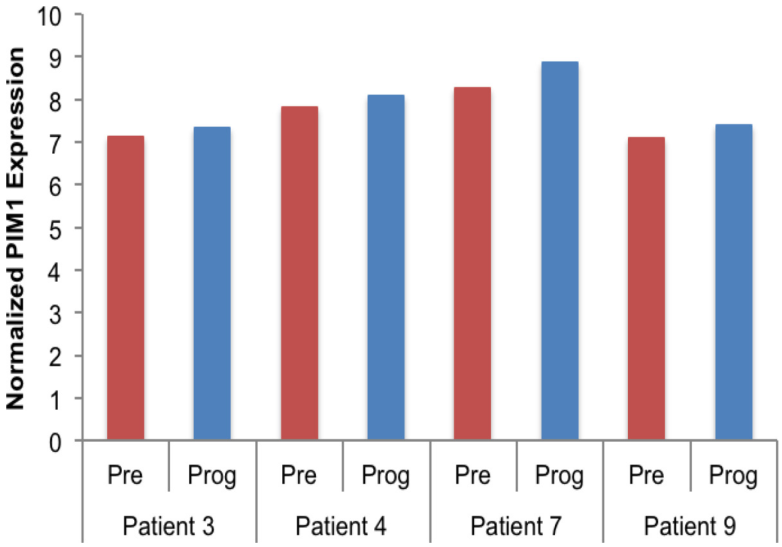

Supplementary Figure S3: PIM1 gene expression in melanoma tumors treated with a combination of dabrafenib and trametinib (pre-treatment and upon progression). RNA was isolated from fresh frozen melanoma tumors in 9 patients (GSE61992) using. The graph shows data for 4 patients with greater PIM1 gene expression in progressing tumor samples (Prog) than in pretreated samples (Pre). The y-axis is the normalized PIM1 gene expression level.

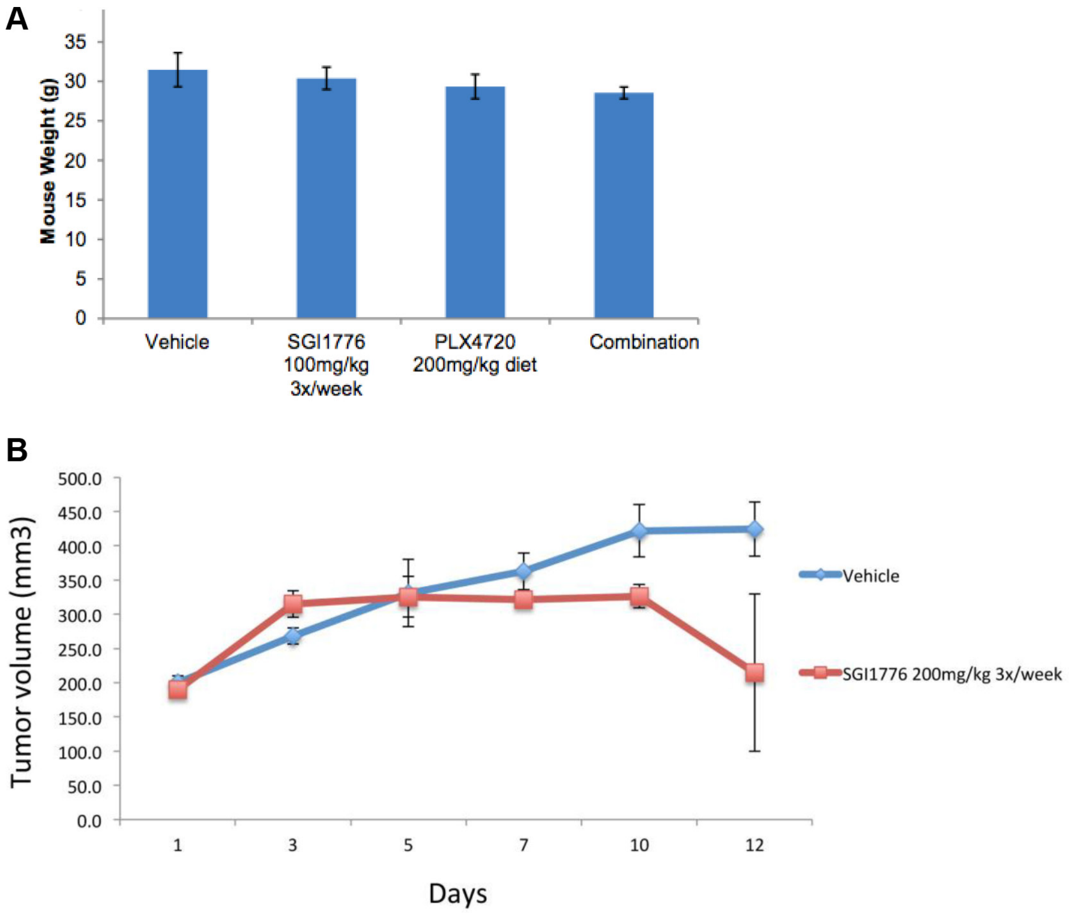

Supplementary Figure S4: SGI-1776 is effective as a single agent or in combination with PLX4720 *in vivo* melanoma models. (**A**) SGI-1776 has minimal effects on total mouse weight as a single agent or in combination with PLX4720. Overall mouse weights from the *in vivo* experiment shown in Figure 6A, measured on the last treatment day. Data are represented as mean +/– SEM. (**B**) SGI-1776 as a single agent is effective in reducing tumor volumes. NSG mice (n = 7/group) were xenotransplanted with 1205Lu melanoma cells and tumors were allowed to grow to 200 mm^3. Mice were then treated with the single agent SGI-1776 (200 mg/kg 3x/week). Tumor volumes were measured at the indicated time points and are represented as mean +/– SEM.

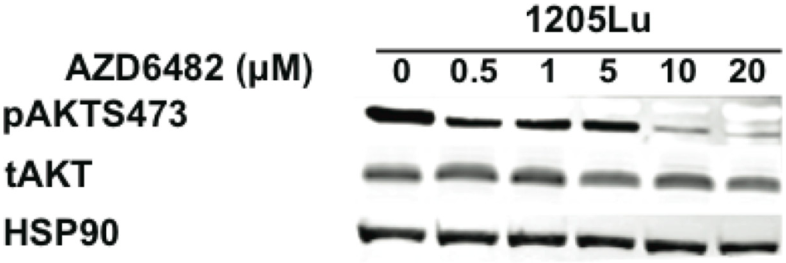

Supplementary Figure S5: AZD6482 target inhibition. Increasing doses of the PI3Kβ inhibitor AZD6482 were used to treat melanoma cells for 72 h before lysates were collected and probed for pAKTSer473 and total protein. Hsp90 was used as a loading control.

1205Lu

SGI-1776 (columns); MK2206 (rows)

	DMSO	1uM	5uM	10uM
DMSO	100	91.7	5.64	6.65
1uM	75.41	56.83	4.43	5.25
5uM	54.1	43.25	4.57	6.41
10uM	36.8	17.56	4.8	5.93

Excess over drug additive effects

	1uM	5uM	10uM
1uM	12	0	0
5uM	6	-2	-3
10uM	16	-3	-3

WM983B

SGI-1776 (columns); MK2206 (rows)

	DMSO	1uM	5uM	10uM
DMSO	100	72.96	39.87	47.22
1uM	70.45	28.46	19.15	26.11
5uM	46.37	26.13	18.63	24
10uM	50.53	37.38	24.12	27.65

Excess over drug additive effects

	1uM	5uM	10uM
1uM	23	9	7
5uM	8	0	-2
10uM	-1	-4	-4

1205Lu

SGI-1776 (columns); UO126 (rows)

	DMSO	1uM	5uM	10uM
DMSO	100	67.21	6.37	4.76
1uM	70.91	45.82	4.57	4.38
5uM	50.86	25.83	3.84	5.16
10uM	39.72	18.83	4.62	6.54

Excess over drug additive effects

	1uM	5uM	10uM
1uM	2	0	-1
5uM	8	-1	-3
10uM	8	-2	-5

WM983B

SGI-1776 (columns); UO126 (rows)

	DMSO	1uM	5uM	10uM
DMSO	100	78.66	48.72	53.27
1uM	80.08	46.73	21.84	41.19
5uM	62.95	38.41	20.48	38.82
10uM	74.85	47.97	30.24	47.54

Excess over drug additive effects

	1uM	5uM	10uM
1uM	16	17	1
5uM	11	10	-5
10uM	11	6	-8

Supplementary Figure S6: SGI-1776 displays anti-melanoma activity in combination with an AKT or MEK inhibitor. Melanoma cells were treated for 72 h with SGI-1776 or the AKT inhibitor MK2206 or the MEK inhibitor UO126 as single agents or in combination. To facilitate statistical analysis of synergy effects, we used a grid-like design of constant ratio drug combinations. Viability was calculated relative to DMSO controls in each plate and the mean of three experiments provided. Synergy was calculated for combination experiments using the Bliss formula. The Bliss number gives the difference between predicted and observed inhibition values (excess over Bliss); a positive value indicates synergy, a negative value indicates antagonism and values near zero indicate an overlap of predicted and observed combination effects.

Screened organometallic compounds

The compounds were synthesized according to published procedures [1–5].

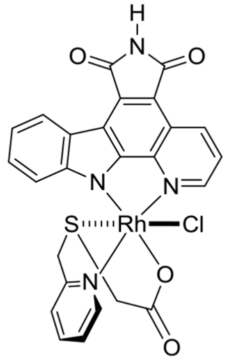

SM407-1

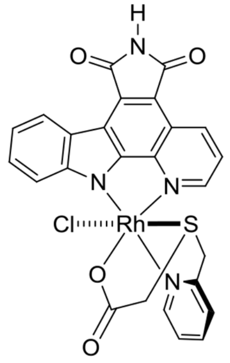

SM407-2

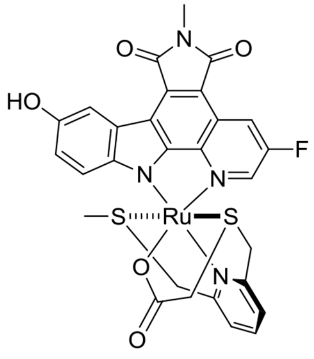

SM250-2

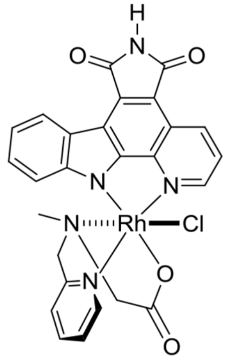

SM406-1

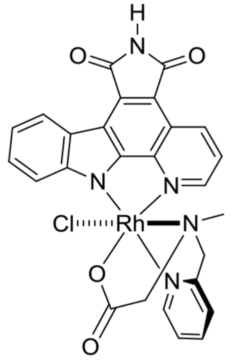

SM406-2

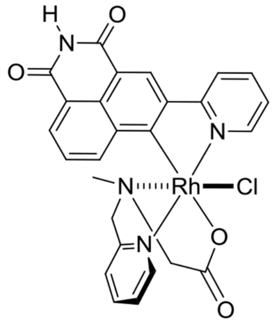

SM426-2

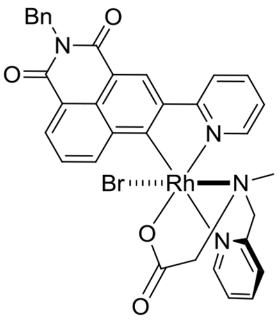

SM439-2

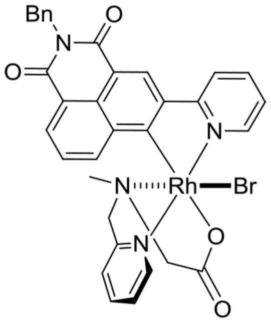

SM439-3

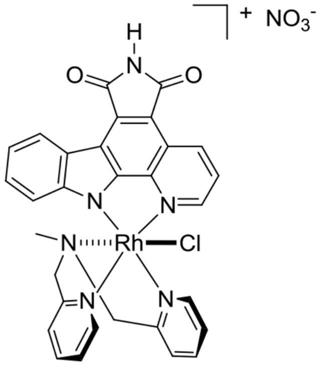

SM449

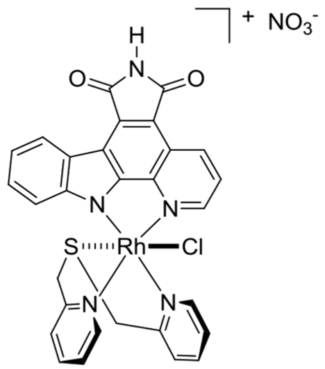

SM450

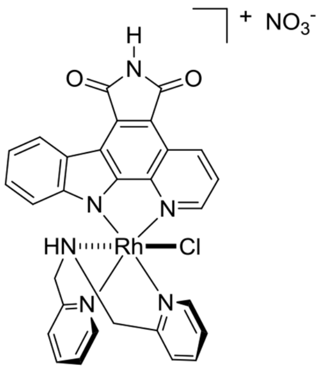

SM451

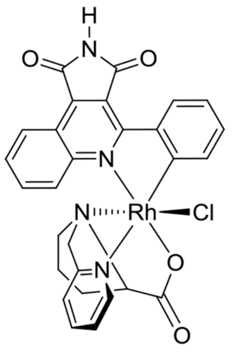

SM440-1

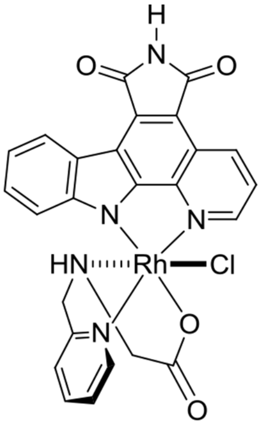

SM415-1

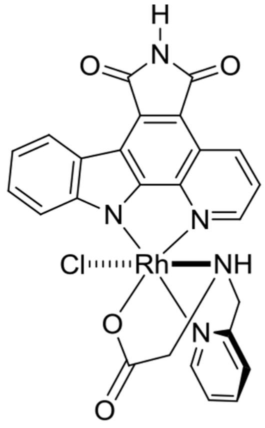

SM415-2

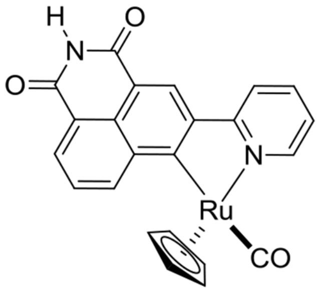

SB_TC-18

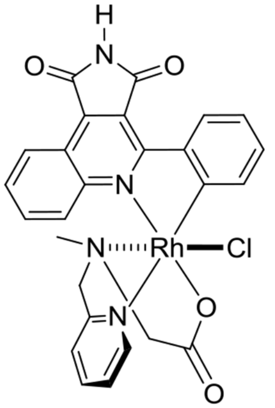

SM438-2

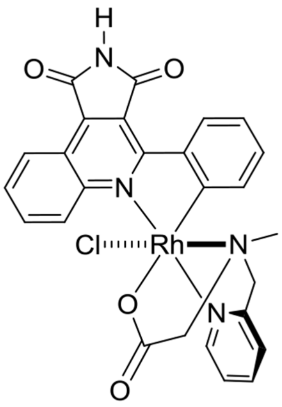

SM438-3

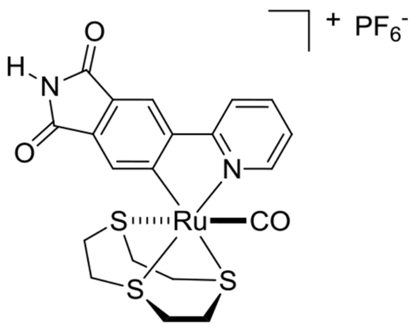

SB_178

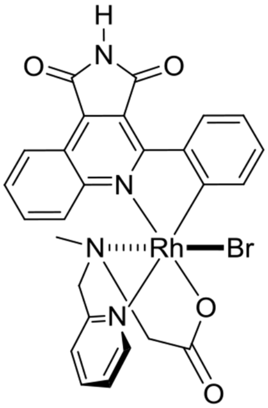

SM440-1

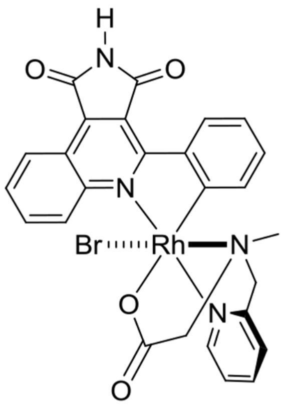

SM440-3

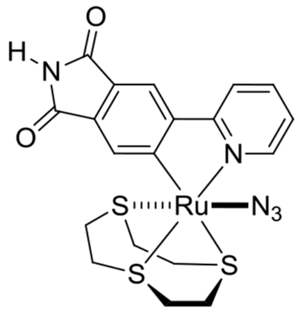

SB_179

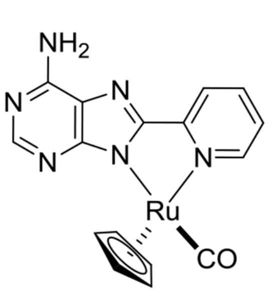

MCS_C002

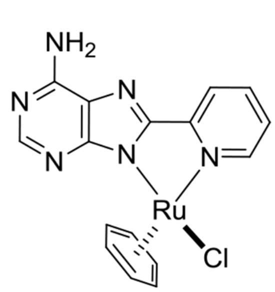

MCS_C003

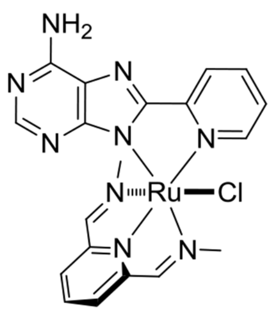

MCS_C010

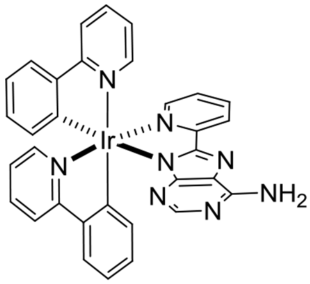

MCS_C007

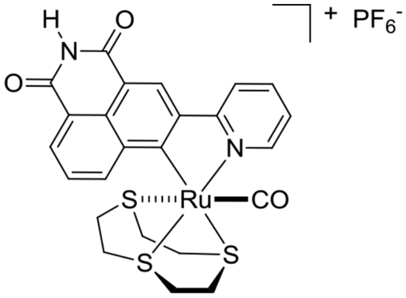

SB_181

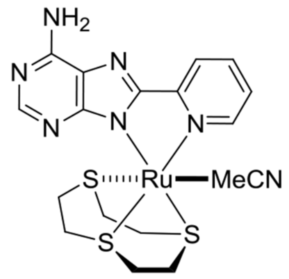

MCS_C004

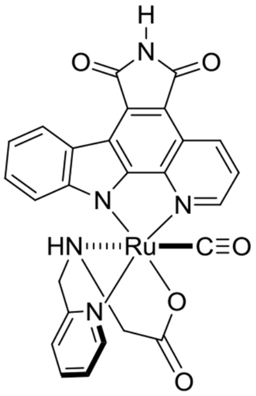

SM200-1 (SM200)

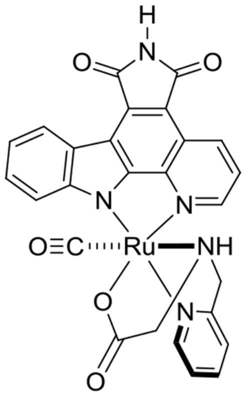

SM200-2

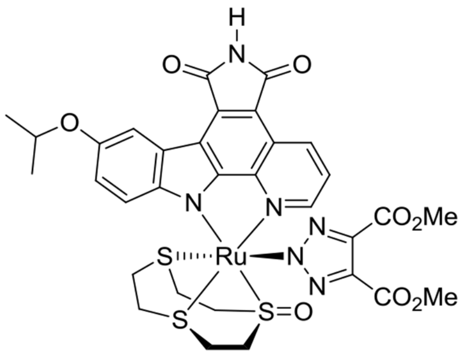

FL1543

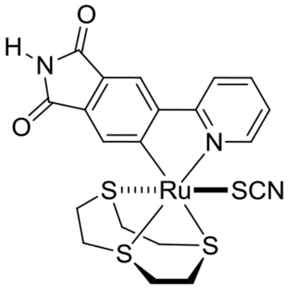

SB_171

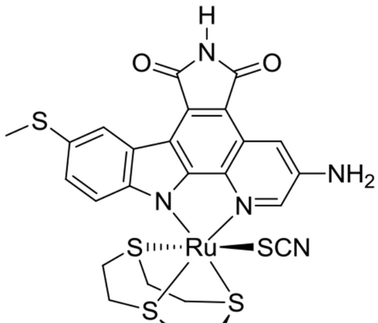

FL1334

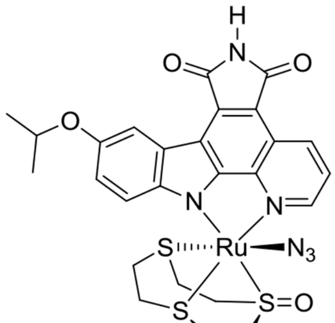

FL1528-1

Synthesis of SM200

The Ru(II)-precursor 1 [6] and *N*-(2-pyridylmethyl) glycine 2 [7] were synthesized according to reported methods. Et_3N was used without further purification and DMF was distilled over CaH_2 before usage. TLC plates for thin layer chromatography (*silica gel 60 F_{254}*) and silica gel for column chromatography (*silica gel 60*, 40–63 µm) were supplied by *Merck KGaA*. ^{1}H-NMR spectra were recorded using a *Bruker Avance 300* (300 MHz), for IR-spectra a *Bruker Alpha-P* FT-IR spectrometer and for mass spectra a *LTQ-FT Ultra* mass spectrometer from *Thermo Fisher Scientific*.

A solution of Ru(II)-precursor 1 (10.9 mg, 20.0 µmol) in 2.0 µL anhydrous DMF was heated to 85°C under an atmosphere of CO. After 90 min, Et_3N (8.4 mL, 60.0 µmol) and *N*-(2-pyridylmethyl)glycine 2 (3.7 mg, 20.0 µmol) were added to the red solution and stirred again at 85°C for 90 min. The red solution was cooled to room temperature, all volatile compounds were evaporated in vacuo and the mixture subjected to column chromatography (CH_2Cl_2/ MeOH 50:1–30:1). After purification, 1.0 mg (1.72 µmol, 9%) of SM200 and 1.1 mg (1.89 µmol, 9%) of SM200-2 were isolated as red solids.

SM200: R_f = 0.18 (CH_2Cl_2/MeOH 20:1). ^{1}H-NMR (300 MHz, DMSO-*d6*): δ (ppm) = 11.15 (s, 1H, CH_{ar}), 9.34 (d, J = 5.3 Hz, 1H, CH_{ar}), 9.20 (d, J = 7.3 Hz, 1H, CH_{ar}), 8.67–8.62 (m, 2H, CH_{ar}), 8.13 (td, J = 7.8 Hz, 1.4 Hz, 1H, CH_{ar}), 7.92 (dd, J = 8.4, 5.1 Hz, 1H, CH_{ar}), 7.72–7.68 (m, 2H, CH_{ar}), 7.25-7.15 (m, 2H, CH_{ar}), 6.55 (s, 1H, CH_{ar}), 5.95 (d, J = 6.5 Hz, 1H, CH_{ar}), 4.32 (d, J = 16.4 Hz, 1H, NCH_2), 4.00 (dd, J = 16.5, 5.6 Hz, 1H, NCH_2), 3.58 (dd, J = 17.2, 8.2 Hz, 1H, NCH_2), 3.18-3.13 (m, 1H, NCH_2). IR: n (cm^{-1}) 3415, 3241, 2924, 1948, 1747, 1701, 1612, 1422, 1346, 1231, 748, 643. HRMS (ESI(+)): $C_{26}H_{18}N_5O_5Ru$ $(M+H)^+$ calc. 582.0353, found 582.0342.

SM200-2: R_f = 0.13 (CH_2Cl_2/MeOH 20:1). ^{1}H-NMR (300 MHz, DMSO-*d6*): δ (ppm) = 11.13 (s, 1H, CH_{ar}), 9.21 (d, J = 5.4 Hz, 1H, CH_{ar}), 9.11 (d, J = 8.4, 1.2 Hz, 1H, CH_{ar}), 8.70 (d, J = 7.9 Hz, 1H, CH_{ar}), 8.08-8.02 (m, 2H, CH_{ar}), 7.69–7.54 (m, 4H, CH_{ar}), 7.47 (td, J = 8.2, 1.3 Hz, 1H, CH_{ar}), 7.35–7.30 (m, 1H, CH_{ar}), 6.55 (s, 1H, CH_{ar}), 4.43-4.27 (m, 2H, NCH_2), 3.28-3.24 (m, 2H, NCH_2). IR: n (cm^{-1}) 3382, 1942, 1689, 1629, 1292, 1150, 1021, 817, 732, 703, 481, 424. HRMS (ESI(+)): $C_{26}H_{18}N_5O_5Ru$ $(M+H)^+$ calc. 582.0353, found 582.0341.

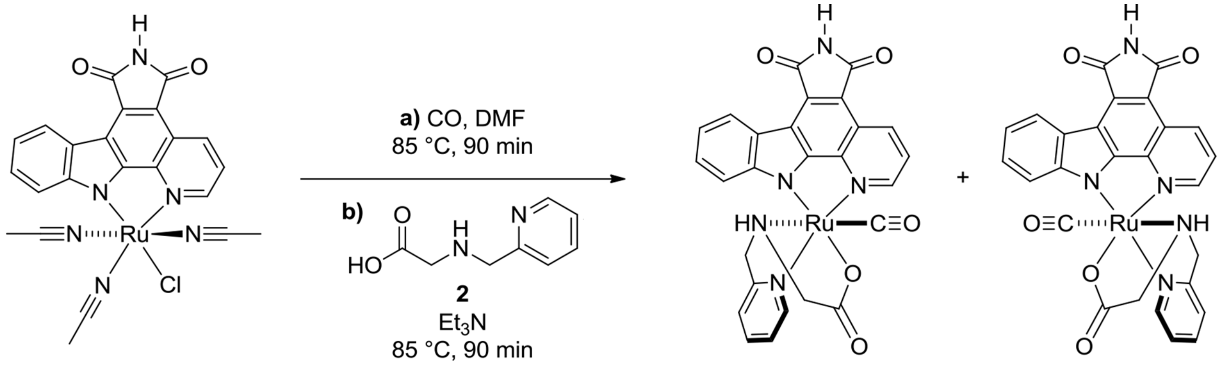

1 SM200 SM200-2

Flow cytometry staining

Caspase-3 activated intracellular staining was achieved after cell fixation and permeabilization (BD Cytofix/Cytoperm™ Fixation/Permeabilization Solution Kit), according to manufacturer's instructions. Cells were stained with primary Purified Rabbit anti-human caspase-3 activated antibody (Cell Signaling Technologies) diluted 1:200, 30 min at 4°C. This was followed by a secondary antibody incubation using anti-mouse Alexa488 (Life Technologies), 1:500 dilution, 20 min at 4°C. The percent of positive cells after SM200 treatment are reported after subtraction of background from the control staining. Samples were acquired with a Becton Dickinson LSRII 14 color flow cytometer and analyzed with FlowJo software (Tree Star Inc. Ashland, OR, USA).

Supplementary Table S1: Melanoma cell line basic genetic information

Cell Line	Stage	BRAF	NRAS
**WM3918**	MET	WT	WT
**WM983B**<br>**WM983B-BR**	MET	V600E	WT
**1205Lu**	MET	V600E	WT
**451Lu**<br>**451Lu-BR**	MET	V600E	WT
**WM1361A**	VGP	WT	Q61R

Stage and BRAF, NRAS mutation status is provided.
Abbreviations: MET, metastasis; WT, wild type; N/A, not available; BR, BRAF inhibitor resistant cell line.

Supplementary Table S2: Organometallic compound effects on melanoma cell lines

Compounds with no anti-melanoma activity	Compounds selective for melanoma cells (low dose)	Compounds selective for melanoma cells (higher dose)	Cytotoxic compounds
IC50 > 10 μM	IC50 ≤ 10 μM	IC50 > 10 μM	IC50 ≥ 5 μM
SB_178<br>SB_TC-18<br>MCS_C003<br>MCS_C010<br>SM250-2<br>SM406-1, -2<br>SM407-1, -2<br>SM415-1, -2<br>SM426-2<br>SM438-2, -3<br>SM439-2, -3<br>SM440-1, -3<br>SM449<br>SM450<br>SM451	SM200<br>SM200-2<br>FL1543	SB_179<br>SB_171<br>MCS_C002<br>MCS_C007	MCS_C004<br>SB_181<br>FL1528-01<br>FL1334

Compounds were tested on fibroblasts (control) and the melanoma cell lines 1205Lu, 451Lu, WM983B, and WM3918 over 72 h; effects were assessed using the MTS assay.

Supplementary Table S3: Kinase profiling of SM200. See Supplementary_Table_S3

REFERENCES

1. Bregman H, Carroll PJ, Meggers E. J Am Chem Soc. 2006; 128; 877–884.
2. Feng L, Geisselbrecht Y, Blanck S, Wilbuer A, Atilla-Gokcumen GE, Filippakopoulos P, Kräling K, Celik MA, Harms K, Maksimoska J, Marmorstein R, Frenking G, Knapp S. J Am Chem Soc. 2011; 133:5976–5986.
3. Blanck S, Cruchter T, Vultur A, Riedel R, Harms K, Herlyn M, Meggers E. Organometallics. 2011; 30:4598–4606.
4. Mollin S, Blanck S, Harms K, Meggers E. Inorg Chim Acta 2012; 393:261–268.
5. Streib M, Kräling K, Richter K, Xie X, Steuber H, Meggers E. Angew Chem Int Ed. 2014; 53:305–309.
6. Bregman H, Carroll PJ, Meggers. E J Am Chem Soc. 2006; 128:877–884.
7. Incarvito C, Rheingold AL, Gavriola AL, Qin CJ, Bosnich B. Inorg Chem. 2001; 40:4101–4108.
8. Fabian MA, Biggs WH III, Treiber DK, Atteridge CE, Azimoara MD, Benedetti MG, Carter TA, Ciceri P, Edeen PT, Floyd M, Ford JM, Galvin M, Gerlach JL. Nat Biotechnol. 2005; 23:329–36; (b). Karaman MW, Herrgard S, Treiber DK, Gallant P, Atteridge CE, Campbell BT, Chan KW, Ciceri P, Davis MI, Edeen PT, Faraoni R, Floyd M, Hunt JP. Nat Biotechnol. 2008; 26:127–132.
