# Supplementary material for: PIM kinases as therapeutic targets against advanced melanoma
Source: Oncotarget. 2016 Jul 19;7(34):54897–912. doi: 10.18632/oncotarget.10703 (PMC5342389; doi:10.18632/oncotarget.10703)
Supplement: Supplementary file 2 [file oncotarget-07-54897-s002.docx]

| **Ambit Gene Symbol** | **Entrez Gene Symbol** | **% Ctrl** |
| --- | --- | --- |
| AAK1 | AAK1 | 96 |
| ABL1(E255K)-phosphorylated | ABL1 | 83 |
| ABL1(F317I)-nonphosphorylated | ABL1 | 100 |
| ABL1(F317I)-phosphorylated | ABL1 | 80 |
| ABL1(F317L)-nonphosphorylated | ABL1 | 88 |
| ABL1(F317L)-phosphorylated | ABL1 | 74 |
| ABL1(H396P)-nonphosphorylated | ABL1 | 61 |
| ABL1(H396P)-phosphorylated | ABL1 | 84 |
| ABL1(M351T)-phosphorylated | ABL1 | 74 |
| ABL1(Q252H)-nonphosphorylated | ABL1 | 74 |
| ABL1(Q252H)-phosphorylated | ABL1 | 100 |
| ABL1(T315I)-nonphosphorylated | ABL1 | 84 |
| ABL1(T315I)-phosphorylated | ABL1 | 85 |
| ABL1(Y253F)-phosphorylated | ABL1 | 88 |
| ABL1-nonphosphorylated | ABL1 | 84 |
| ABL1-phosphorylated | ABL1 | 87 |
| ABL2 | ABL2 | 100 |
| ACVR1 | ACVR1 | 89 |
| ACVR1B | ACVR1B | 100 |
| ACVR2A | ACVR2A | 95 |
| ACVR2B | ACVR2B | 100 |
| ACVRL1 | ACVRL1 | 97 |
| ADCK3 | CABC1 | 85 |
| ADCK4 | ADCK4 | 67 |
| AKT1 | AKT1 | 93 |
| AKT2 | AKT2 | 93 |
| AKT3 | AKT3 | 76 |
| ALK | ALK | 95 |
| AMPK-alpha1 | PRKAA1 | 100 |
| AMPK-alpha2 | PRKAA2 | 81 |
| ANKK1 | ANKK1 | 80 |
| ARK5 | NUAK1 | 95 |
| ASK1 | MAP3K5 | 83 |
| ASK2 | MAP3K6 | 85 |
| AURKA | AURKA | 81 |
| AURKB | AURKB | 68 |
| AURKC | AURKC | 67 |
| AXL | AXL | 79 |
| BIKE | BMP2K | 75 |
| BLK | BLK | 47 |
| BMPR1A | BMPR1A | 92 |
| BMPR1B | BMPR1B | 61 |
| BMPR2 | BMPR2 | 98 |
| BMX | BMX | 76 |
| BRAF | BRAF | 58 |
| BRAF(V600E) | BRAF | 37 |
| BRK | PTK6 | 99 |
| BRSK1 | BRSK1 | 93 |
| BRSK2 | BRSK2 | 68 |
| BTK | BTK | 95 |
| BUB1 | BUB1 | 83 |
| CAMK1 | CAMK1 | 72 |
| CAMK1D | CAMK1D | 80 |
| CAMK1G | CAMK1G | 92 |
| CAMK2A | CAMK2A | 62 |
| CAMK2B | CAMK2B | 86 |
| CAMK2D | CAMK2D | 93 |
| CAMK2G | CAMK2G | 96 |
| CAMK4 | CAMK4 | 100 |
| CAMKK1 | CAMKK1 | 74 |
| CAMKK2 | CAMKK2 | 100 |
| CASK | CASK | 91 |
| CDC2L1 | CDK11B | 100 |
| CDC2L2 | CDC2L2 | 100 |
| CDC2L5 | CDK13 | 77 |
| CDK11 | CDK19 | 90 |
| CDK2 | CDK2 | 95 |
| CDK3 | CDK3 | 100 |
| CDK4-cyclinD1 | CDK4 | 67 |
| CDK4-cyclinD3 | CDK4 | 100 |
| CDK5 | CDK5 | 100 |
| CDK7 | CDK7 | 85 |
| CDK8 | CDK8 | 68 |
| CDK9 | CDK9 | 100 |
| CDKL1 | CDKL1 | 100 |
| CDKL2 | CDKL2 | 68 |
| CDKL3 | CDKL3 | 100 |
| CDKL5 | CDKL5 | 85 |
| CHEK1 | CHEK1 | 77 |
| CHEK2 | CHEK2 | 86 |
| CIT | CIT | 70 |
| CLK1 | CLK1 | 32 |
| CLK2 | CLK2 | 5.2 |
| CLK3 | CLK3 | 82 |
| CLK4 | CLK4 | 18 |
| CSF1R | CSF1R | 100 |
| CSF1R-autoinhibited | CSF1R | 85 |
| CSK | CSK | 71 |
| CSNK1A1 | CSNK1A1 | 83 |
| CSNK1A1L | CSNK1A1L | 77 |
| CSNK1D | CSNK1D | 91 |
| CSNK1E | CSNK1E | 90 |
| CSNK1G1 | CSNK1G1 | 90 |
| CSNK1G2 | CSNK1G2 | 83 |
| CSNK1G3 | CSNK1G3 | 91 |
| CSNK2A1 | CSNK2A1 | 74 |
| CSNK2A2 | CSNK2A2 | 41 |
| CTK | MATK | 55 |
| DAPK1 | DAPK1 | 49 |
| DAPK2 | DAPK2 | 25 |
| DAPK3 | DAPK3 | 49 |
| DCAMKL1 | DCLK1 | 63 |
| DCAMKL2 | DCLK2 | 100 |
| DCAMKL3 | DCLK3 | 98 |
| DDR1 | DDR1 | 88 |
| DDR2 | DDR2 | 100 |
| DLK | MAP3K12 | 100 |
| DMPK | DMPK | 93 |
| DMPK2 | CDC42BPG | 76 |
| DRAK1 | STK17A | 84 |
| DRAK2 | STK17B | 86 |
| DYRK1A | DYRK1A | 23 |
| DYRK1B | DYRK1B | 50 |
| DYRK2 | DYRK2 | 69 |
| EGFR | EGFR | 94 |
| EGFR(E746-A750del) | EGFR | 69 |
| EGFR(G719C) | EGFR | 79 |
| EGFR(G719S) | EGFR | 82 |
| EGFR(L747-E749del, A750P) | EGFR | 81 |
| EGFR(L747-S752del, P753S) | EGFR | 97 |
| EGFR(L747-T751del,Sins) | EGFR | 71 |
| EGFR(L858R) | EGFR | 84 |
| EGFR(L858R,T790M) | EGFR | 80 |
| EGFR(L861Q) | EGFR | 87 |
| EGFR(S752-I759del) | EGFR | 98 |
| EGFR(T790M) | EGFR | 48 |
| EIF2AK1 | EIF2AK1 | 92 |
| EPHA1 | EPHA1 | 67 |
| EPHA2 | EPHA2 | 95 |
| EPHA3 | EPHA3 | 74 |
| EPHA4 | EPHA4 | 100 |
| EPHA5 | EPHA5 | 100 |
| EPHA6 | EPHA6 | 100 |
| EPHA7 | EPHA7 | 100 |
| EPHA8 | EPHA8 | 100 |
| EPHB1 | EPHB1 | 84 |
| EPHB2 | EPHB2 | 82 |
| EPHB3 | EPHB3 | 100 |
| EPHB4 | EPHB4 | 100 |
| EPHB6 | EPHB6 | 55 |
| ERBB2 | ERBB2 | 72 |
| ERBB3 | ERBB3 | 67 |
| ERBB4 | ERBB4 | 97 |
| ERK1 | MAPK3 | 88 |
| ERK2 | MAPK1 | 100 |
| ERK3 | MAPK6 | 80 |
| ERK4 | MAPK4 | 100 |
| ERK5 | MAPK7 | 100 |
| ERK8 | MAPK15 | 37 |
| ERN1 | ERN1 | 61 |
| FAK | PTK2 | 100 |
| FER | FER | 77 |
| FES | FES | 100 |
| FGFR1 | FGFR1 | 90 |
| FGFR2 | FGFR2 | 70 |
| FGFR3 | FGFR3 | 72 |
| FGFR3(G697C) | FGFR3 | 50 |
| FGFR4 | FGFR4 | 100 |
| FGR | FGR | 100 |
| FLT1 | FLT1 | 90 |
| FLT3 | FLT3 | 50 |
| FLT3(D835H) | FLT3 | 18 |
| FLT3(D835Y) | FLT3 | 14 |
| FLT3(ITD) | FLT3 | 43 |
| FLT3(K663Q) | FLT3 | 23 |
| FLT3(N841I) | FLT3 | 9.2 |
| FLT3(R834Q) | FLT3 | 34 |
| FLT3-autoinhibited | FLT3 | 87 |
| FLT4 | FLT4 | 83 |
| FRK | FRK | 100 |
| FYN | FYN | 100 |
| GAK | GAK | 85 |
| GCN2(Kin.Dom.2,S808G) | EIF2AK4 | 97 |
| GRK1 | GRK1 | 29 |
| GRK4 | GRK4 | 74 |
| GRK7 | GRK7 | 8.8 |
| GSK3A | GSK3A | 9.4 |
| GSK3B | GSK3B | 26 |
| HASPIN | GSG2 | 72 |
| HCK | HCK | 81 |
| HIPK1 | HIPK1 | 3.6 |
| HIPK2 | HIPK2 | 2.4 |
| HIPK3 | HIPK3 | 2.6 |
| HIPK4 | HIPK4 | 68 |
| HPK1 | MAP4K1 | 77 |
| HUNK | HUNK | 70 |
| ICK | ICK | 75 |
| IGF1R | IGF1R | 94 |
| IKK-alpha | CHUK | 91 |
| IKK-beta | IKBKB | 100 |
| IKK-epsilon | IKBKE | 64 |
| INSR | INSR | 74 |
| INSRR | INSRR | 85 |
| IRAK1 | IRAK1 | 0.2 |
| IRAK3 | IRAK3 | 49 |
| IRAK4 | IRAK4 | 70 |
| ITK | ITK | 100 |
| JAK1(JH1domain-catalytic) | JAK1 | 94 |
| JAK1(JH2domain-pseudokinase) | JAK1 | 78 |
| JAK2(JH1domain-catalytic) | JAK2 | 71 |
| JAK3(JH1domain-catalytic) | JAK3 | 23 |
| JNK1 | MAPK8 | 100 |
| JNK2 | MAPK9 | 100 |
| JNK3 | MAPK10 | 100 |
| KIT | KIT | 70 |
| KIT(A829P) | KIT | 52 |
| KIT(D816H) | KIT | 80 |
| KIT(D816V) | KIT | 70 |
| KIT(L576P) | KIT | 73 |
| KIT(V559D) | KIT | 61 |
| KIT(V559D,T670I) | KIT | 95 |
| KIT(V559D,V654A) | KIT | 81 |
| KIT-autoinhibited | KIT | 96 |
| LATS1 | LATS1 | 83 |
| LATS2 | LATS2 | 63 |
| LCK | LCK | 71 |
| LIMK1 | LIMK1 | 87 |
| LIMK2 | LIMK2 | 92 |
| LKB1 | STK11 | 100 |
| LOK | STK10 | 100 |
| LRRK2 | LRRK2 | 82 |
| LRRK2(G2019S) | LRRK2 | 86 |
| LTK | LTK | 100 |
| LYN | LYN | 72 |
| LZK | MAP3K13 | 93 |
| MAK | MAK | 73 |
| MAP3K1 | MAP3K1 | 89 |
| MAP3K15 | MAP3K15 | 100 |
| MAP3K2 | MAP3K2 | 100 |
| MAP3K3 | MAP3K3 | 96 |
| MAP3K4 | MAP3K4 | 64 |
| MAP4K2 | MAP4K2 | 88 |
| MAP4K3 | MAP4K3 | 100 |
| MAP4K4 | MAP4K4 | 94 |
| MAP4K5 | MAP4K5 | 91 |
| MAPKAPK2 | MAPKAPK2 | 91 |
| MAPKAPK5 | MAPKAPK5 | 100 |
| MARK1 | MARK1 | 85 |
| MARK2 | MARK2 | 42 |
| MARK3 | MARK3 | 83 |
| MARK4 | MARK4 | 100 |
| MAST1 | MAST1 | 100 |
| MEK1 | MAP2K1 | 85 |
| MEK2 | MAP2K2 | 83 |
| MEK3 | MAP2K3 | 81 |
| MEK4 | MAP2K4 | 100 |
| MEK5 | MAP2K5 | 90 |
| MEK6 | MAP2K6 | 71 |
| MELK | MELK | 77 |
| MERTK | MERTK | 84 |
| MET | MET | 97 |
| MET(M1250T) | MET | 90 |
| MET(Y1235D) | MET | 88 |
| MINK | MINK1 | 83 |
| MKK7 | MAP2K7 | 76 |
| MKNK1 | MKNK1 | 87 |
| MKNK2 | MKNK2 | 93 |
| MLCK | MYLK3 | 78 |
| MLK1 | MAP3K9 | 69 |
| MLK2 | MAP3K10 | 84 |
| MLK3 | MAP3K11 | 72 |
| MRCKA | CDC42BPA | 93 |
| MRCKB | CDC42BPB | 100 |
| MST1 | STK4 | 96 |
| MST1R | MST1R | 77 |
| MST2 | STK3 | 77 |
| MST3 | STK24 | 96 |
| MST4 | MST4 | 71 |
| MTOR | MTOR | 75 |
| MUSK | MUSK | 100 |
| MYLK | MYLK | 2 |
| MYLK2 | MYLK2 | 100 |
| MYLK4 | MYLK4 | 99 |
| MYO3A | MYO3A | 70 |
| MYO3B | MYO3B | 77 |
| NDR1 | STK38 | 98 |
| NDR2 | STK38L | 94 |
| NEK1 | NEK1 | 68 |
| NEK11 | NEK11 | 70 |
| NEK2 | NEK2 | 86 |
| NEK3 | NEK3 | 89 |
| NEK4 | NEK4 | 58 |
| NEK5 | NEK5 | 99 |
| NEK6 | NEK6 | 95 |
| NEK7 | NEK7 | 80 |
| NEK9 | NEK9 | 87 |
| NIM1 | MGC42105 | 70 |
| NLK | NLK | 65 |
| OSR1 | OXSR1 | 91 |
| p38-alpha | MAPK14 | 72 |
| p38-beta | MAPK11 | 99 |
| p38-delta | MAPK13 | 40 |
| p38-gamma | MAPK12 | 67 |
| PAK1 | PAK1 | 95 |
| PAK2 | PAK2 | 37 |
| PAK3 | PAK3 | 100 |
| PAK4 | PAK4 | 59 |
| PAK6 | PAK6 | 41 |
| PAK7 | PAK7 | 31 |
| PCTK1 | CDK16 | 91 |
| PCTK2 | CDK17 | 72 |
| PCTK3 | CDK18 | 100 |
| PDGFRA | PDGFRA | 68 |
| PDGFRB | PDGFRB | 46 |
| PDPK1 | PDPK1 | 84 |
| PFCDPK1(P.falciparum) | CDPK1 | 84 |
| PFPK5(P.falciparum) | MAL13P1.279 | 90 |
| PFTAIRE2 | CDK15 | 81 |
| PFTK1 | CDK14 | 100 |
| PHKG1 | PHKG1 | 78 |
| PHKG2 | PHKG2 | 100 |
| PIK3C2B | PIK3C2B | 88 |
| PIK3C2G | PIK3C2G | 98 |
| PIK3CA | PIK3CA | 100 |
| PIK3CA(C420R) | PIK3CA | 84 |
| PIK3CA(E542K) | PIK3CA | 79 |
| PIK3CA(E545A) | PIK3CA | 98 |
| PIK3CA(E545K) | PIK3CA | 78 |
| PIK3CA(H1047L) | PIK3CA | 88 |
| PIK3CA(H1047Y) | PIK3CA | 81 |
| PIK3CA(I800L) | PIK3CA | 85 |
| PIK3CA(M1043I) | PIK3CA | 61 |
| PIK3CA(Q546K) | PIK3CA | 100 |
| PIK3CB | PIK3CB | 82 |
| PIK3CD | PIK3CD | 60 |
| PIK3CG | PIK3CG | 96 |
| PIK4CB | PI4KB | 91 |
| PIM1 | PIM1 | 3.5 |
| PIM2 | PIM2 | 46 |
| PIM3 | PIM3 | 6.4 |
| PIP5K1A | PIP5K1A | 100 |
| PIP5K1C | PIP5K1C | 68 |
| PIP5K2B | PIP4K2B | 100 |
| PIP5K2C | PIP4K2C | 50 |
| PKAC-alpha | PRKACA | 82 |
| PKAC-beta | PRKACB | 86 |
| PKMYT1 | PKMYT1 | 100 |
| PKN1 | PKN1 | 49 |
| PKN2 | PKN2 | 46 |
| PKNB(M.tuberculosis) | pknB | 71 |
| PLK1 | PLK1 | 90 |
| PLK2 | PLK2 | 76 |
| PLK3 | PLK3 | 68 |
| PLK4 | PLK4 | 40 |
| PRKCD | PRKCD | 46 |
| PRKCE | PRKCE | 62 |
| PRKCH | PRKCH | 58 |
| PRKCI | PRKCI | 100 |
| PRKCQ | PRKCQ | 51 |
| PRKD1 | PRKD1 | 85 |
| PRKD2 | PRKD2 | 100 |
| PRKD3 | PRKD3 | 85 |
| PRKG1 | PRKG1 | 80 |
| PRKG2 | PRKG2 | 3.5 |
| PRKR | EIF2AK2 | 72 |
| PRKX | PRKX | 86 |
| PRP4 | PRPF4B | 83 |
| PYK2 | PTK2B | 62 |
| QSK | KIAA0999 | 79 |
| RAF1 | RAF1 | 83 |
| RET | RET | 100 |
| RET(M918T) | RET | 81 |
| RET(V804L) | RET | 73 |
| RET(V804M) | RET | 74 |
| RIOK1 | RIOK1 | 96 |
| RIOK2 | RIOK2 | 85 |
| RIOK3 | RIOK3 | 79 |
| RIPK1 | RIPK1 | 75 |
| RIPK2 | RIPK2 | 100 |
| RIPK4 | RIPK4 | 100 |
| RIPK5 | DSTYK | 62 |
| ROCK1 | ROCK1 | 89 |
| ROCK2 | ROCK2 | 100 |
| ROS1 | ROS1 | 67 |
| RPS6KA4(Kin.Dom.1-N-terminal) | RPS6KA4 | 95 |
| RPS6KA4(Kin.Dom.2-C-terminal) | RPS6KA4 | 82 |
| RPS6KA5(Kin.Dom.1-N-terminal) | RPS6KA5 | 78 |
| RPS6KA5(Kin.Dom.2-C-terminal) | RPS6KA5 | 100 |
| RSK1(Kin.Dom.1-N-terminal) | RPS6KA1 | 70 |
| RSK1(Kin.Dom.2-C-terminal) | RPS6KA1 | 78 |
| RSK2(Kin.Dom.1-N-terminal) | RPS6KA3 | 51 |
| RSK2(Kin.Dom.2-C-terminal) | RPS6KA3 | 99 |
| RSK3(Kin.Dom.1-N-terminal) | RPS6KA2 | 55 |
| RSK3(Kin.Dom.2-C-terminal) | RPS6KA2 | 90 |
| RSK4(Kin.Dom.1-N-terminal) | RPS6KA6 | 70 |
| RSK4(Kin.Dom.2-C-terminal) | RPS6KA6 | 81 |
| S6K1 | RPS6KB1 | 54 |
| SBK1 | SBK1 | 60 |
| SGK | SGK1 | 55 |
| SgK110 | SgK110 | 85 |
| SGK3 | SGK3 | 70 |
| SIK | SIK1 | 100 |
| SIK2 | SIK2 | 83 |
| SLK | SLK | 58 |
| SNARK | NUAK2 | 64 |
| SNRK | SNRK | 100 |
| SRC | SRC | 100 |
| SRMS | SRMS | 62 |
| SRPK1 | SRPK1 | 70 |
| SRPK2 | SRPK2 | 62 |
| SRPK3 | SRPK3 | 67 |
| STK16 | STK16 | 58 |
| STK33 | STK33 | 82 |
| STK35 | STK35 | 90 |
| STK36 | STK36 | 100 |
| STK39 | STK39 | 60 |
| SYK | SYK | 56 |
| TAK1 | MAP3K7 | 72 |
| TAOK1 | TAOK1 | 84 |
| TAOK2 | TAOK2 | 99 |
| TAOK3 | TAOK3 | 100 |
| TBK1 | TBK1 | 100 |
| TEC | TEC | 78 |
| TESK1 | TESK1 | 47 |
| TGFBR1 | TGFBR1 | 80 |
| TGFBR2 | TGFBR2 | 100 |
| TIE1 | TIE1 | 81 |
| TIE2 | TEK | 100 |
| TLK1 | TLK1 | 84 |
| TLK2 | TLK2 | 84 |
| TNIK | TNIK | 64 |
| TNK1 | TNK1 | 95 |
| TNK2 | TNK2 | 80 |
| TNNI3K | TNNI3K | 100 |
| TRKA | NTRK1 | 76 |
| TRKB | NTRK2 | 74 |
| TRKC | NTRK3 | 81 |
| TRPM6 | TRPM6 | 76 |
| TSSK1B | TSSK1B | 94 |
| TTK | TTK | 65 |
| TXK | TXK | 32 |
| TYK2(JH1domain-catalytic) | TYK2 | 58 |
| TYK2(JH2domain-pseudokinase) | TYK2 | 83 |
| TYRO3 | TYRO3 | 86 |
| ULK1 | ULK1 | 71 |
| ULK2 | ULK2 | 100 |
| ULK3 | ULK3 | 95 |
| VEGFR2 | KDR | 73 |
| VRK2 | VRK2 | 100 |
| WEE1 | WEE1 | 100 |
| WEE2 | WEE2 | 85 |
| WNK1 | WNK1 | 100 |
| WNK3 | WNK3 | 90 |
| YANK1 | STK32A | 100 |
| YANK2 | STK32B | 75 |
| YANK3 | STK32C | 100 |
| YES | YES1 | 84 |
| YSK1 | STK25 | 78 |
| YSK4 | YSK4 | 5.2 |
| ZAK | ZAK | 58 |
| ZAP70 | ZAP70 | 63 |
| The protein kinase selectivity profile of the racemic mixture of SM200 at an assay concentration of 1 µM was derived from an active-site-directed affinity screening against 451 protein kinases (KINOMEscan, DiscoveRx) [8]. | | |
